# Predicting longitudinal basal forebrain volume in the Alzheimer’s disease spectrum: the role of sex and ApoE epsilon 4 genotype

**DOI:** 10.3389/fnins.2026.1730947

**Published:** 2026-02-04

**Authors:** Alice Grazia, Fedor Levin, Frank Jessen, Michael Wagner, Oliver Peters, Josef Priller, Anja Schneider, Jens Wiltfang, Emrah Düzel, Katharina Buerger, Robert Perneczky, Christoph Laske, Annika Spottke, Alfredo Ramirez, Stefan J. Teipel

**Affiliations:** 1 Department of Psychosomatic Medicine, University Medicine Rostock, Rostock, Germany; 2 German Center for Neurodegenerative Diseases (DZNE), Rostock, Germany; 3 German Center for Neurodegenerative Diseases (DZNE), Bonn, Germany; 4 German Center for Neurodegenerative Diseases (DZNE), Berlin, Germany; 5 Charité – Universitätsmedizin Berlin, Institute of Psychiatry and Psychotherapy, Berlin, Germany; 6 Department of Psychiatry and Psychotherapy, Charité, Berlin, Germany; 7 Department of Old Age Psychiatry and Cognitive Disorders, University Hospital Bonn and University of Bonn, Bonn, Germany; 8 German Center for Neurodegenerative Diseases (DZNE), Goettingen, Germany; 9 Department of Psychiatry and Psychotherapy, University Medical Center Goettingen, University of Goettingen, Goettingen, Germany; 10 German Center for Neurodegenerative Diseases (DZNE), Magdeburg, Germany; 11 Institute of Cognitive Neurology and Dementia Research (IKND), Otto-von-Guericke University, Magdeburg, Germany; 12 German Center for Neurodegenerative Diseases (DZNE, Munich), Munich, Germany; 13 Institute for Stroke and Dementia Research (ISD), University Hospital, LMU Munich, Munich, Germany; 14 German Center for Neurodegenerative Diseases (DZNE), Tübingen, Germany; 15 Department of Psychiatry and Psychotherapy, University of Tübingen, Tübingen, Germany; 16 Division of Neurogenetics and Molecular Psychiatry, Department of Psychiatry and Psychotherapy, Faculty of Medicine and University Hospital Cologne, University of Cologne, Cologne, Germany; 17 Department of Psychiatry, Glenn Biggs Institute for Alzheimer’s and Neurodegenerative Diseases, San Antonio, TX, United States; 18 Cologne Excellence Cluster on Cellular Stress Responses in Aging-Associated Disease (CECAD), University of Cologne, Cologne, Germany

**Keywords:** APOE ε4, basal forebrain, hippocampus, homozygotes, sex-differences

## Abstract

**Introduction:**

Imaging studies showed early atrophy of the cholinergic basal forebrain (BF) already at prodromal stages of sporadic Alzheimer’s disease (AD). Women and carriers of the ApoE epsilon 4 (ApoE ε4) allele are more likely to develop the disease; however, the underlying mechanisms are still unclear. Here we aimed at exploring the impact of sex and ApoE ε4 genotype in the AD spectrum on longitudinal measures of the basal forebrain and hippocampus, as a comparison region.

**Methods:**

We leveraged the German multi-centered study DELCODE and analyzed 712 individuals (median age: 71.25 years, interquartile range [IQR] = 9.22) with follow-up MRI scans (median time: 2.8 years, [IQR] = 1.75). Diagnostic groups comprised cognitively normal (*N* = 184), subjective cognitive decline (*N* = 331), mild cognitive impairment (*N* = 128) and AD (*N* = 69). Regarding ApoE genotype, 5% of participants were ε4 homozygotes, while 27% were heterozygotes. Volume segmentation and linear mixed-effect models were used to calculate the effects of ApoE ε4 genotype, sex, diagnosis, age, time and their interactions in TIV-adjusted basal forebrain and hippocampal volumes.

**Results:**

The hippocampus, but not the basal forebrain, showed significant atrophy over time (Hipp: *β* = −0.014, *p* < 0.001; BF: *β* = 0.040, *p* = 0.044). Post-TIV correction, female participants had significantly larger baseline basal forebrain (*β* = 0.300, *p* < 0.001) and hippocampal volumes (*β* = 0.273, *p* < 0.001). ApoE ε4 predicted smaller baseline volumes in both regions. After adjusting for multiple comparisons, faster longitudinal atrophy was observed only for ApoE ε4 homozygotes in the hippocampus (*β* = −0.037, *p* < 0.001), with no corresponding effect in the basal forebrain (*β* = 0.000, *p* = 0.841).

**Discussion:**

Our findings did not show the anticipated longitudinal effects of sex and ApoE ε4 on longitudinal basal forebrain volume. Only hippocampal atrophy progressed significantly faster in ApoE ε4 homozygote carriers. This dissociation may reflect stage-dependent neurodegenerative processes, with early basal forebrain vulnerability followed by more rapid hippocampal decline, as well as methodological and sample-related constraints. If replicated, these findings suggest that hippocampal measures may be more sensitive longitudinal biomarkers in ApoE ε4 homozygotes, while sex- and ApoE ε4-related effects on the cholinergic system may be more prominent at earlier disease stages.

## 1 Introduction

Cross-sectional neuroimaging studies have shown that basal forebrain volume atrophies already at early stages of sporadic Alzheimer’s disease (AD) (Bohnen et al., 2018; Grothe et al., 2012). A functional imaging study in individuals with subjective cognitive decline (SCD) and amyloid pathology found reduced connectivity in the posterior-lateral subdivision of the basal forebrain (Chiesa et al., 2019). Similarly, in a study examining basal forebrain volume reductions in SCD with amyloid pathology (*n* = 24 positive and *n* = 24 negative), results suggested lower volumes for the amyloid-positive group, particularly in total basal forebrain volume (*p* = 0.061) and the Nucleus Basalis subregion (*p* = 0.059), though these differences did not reach significance (Daamen et al., 2023). Another study showed that lower basal forebrain volume was associated with increased tau-PET deposition in widespread cortical regions (Yoo et al., 2024). Recently, we found evidence that basal forebrain volume and global functional connectivity were preserved in asymptomatic carriers of *Presenilin 1 (PS1)* gene mutation for familial AD (Grazia et al., 2025; Teipel et al., 2024a). Longitudinal studies on basal forebrain in sporadic AD report heterogeneous findings. One study has shown that basal forebrain pathology precedes and predicts both entorhinal pathology and memory impairment (Schmitz et al., 2016). Another study showed a consistent effect of hippocampus volume, but not basal forebrain, on longitudinal measures of memory functions (Teipel et al., 2023a, 2023b). A recent study demonstrated that elevated amyloid pathology at baseline, as assessed by PET imaging, did not correlate with accelerated basal forebrain atrophy (Yoo et al., 2024).

Women and carriers of the ApoE ε4 allele are more likely to develop AD (Cavedo et al., 2018). Likewise, ApoE ε4 homozygotes have a higher risk to develop AD compared to ApoE ε4 heterozygotes (Raulin et al., 2022). In addition, women carriers of two copies of the ApoE ε4 allele exhibit more severe amyloid and tau pathology (Corder et al., 2004), worse cognitive decline (Beydoun et al., 2004; Lin et al., 2015) and worse atrophy (Fleisher et al., 2005; Sampedro et al., 2015). However, the mechanisms underlying the role of ApoE ε4 in the pathogenesis of the disease as a whole and in relation to biological sex are still unknown (Gamache et al., 2020). Epidemiological studies consistently report that women have a higher lifetime risk of developing AD compared to men (Cavedo et al., 2018; Gamache et al., 2020). In particular, women in their 60s exhibit a significantly faster age-related cognitive decline than men, as well as greater deterioration in memory and executive function (Cavedo et al., 2018). Moreover, late-onset AD (LOAD) is more prevalent in women, and female patients with LOAD tend to experience more rapid cognitive decline and exhibit greater neuropathological burden than their male counterparts (Gamache et al., 2020). Despite these well-documented sex differences, the reasons for the increased prevalence and age-dependent occurrence of AD in women remain poorly understood (Cavedo et al., 2018). In a previous study looking at basal forebrain and its associations with sex and ApoE ε4, no effect of sex was found on basal forebrain volume (Cavedo et al., 2018). In contrast, another study found significant differences in basal forebrain volume between MCI and healthy controls only in the female group (Shi et al., 2024). In our previous studies on asymptomatic PS1 carriers, we found no effect of sex and ApoE ε4 genotype (Grazia et al., 2025; Teipel et al., 2024a).

These findings underscore the limited understanding of the pathophysiological mechanisms underlying AD, particularly those involved in the degeneration of the cholinergic system, which plays a critical role in cognitive function. They highlight the scarcity of longitudinal studies investigating progressive basal forebrain atrophy in sporadic and genetically driven AD. Moreover, the differences observed between the sporadic and familial forms of AD with respect to the basal forebrain raise the question of which other biological and genetic factors might contribute to the vulnerability of the cholinergic system in sporadic forms. In this study, we aimed to investigate the impact of sex and ApoE ε4 genotype on longitudinal measures of basal forebrain and hippocampal volume along the sporadic AD spectrum. We choose the hippocampus as a comparison region given its well-established involvement in early AD pathology and its known vulnerability to amyloid deposition and volumes changes (Grothe et al., 2012). Our primary hypothesis was that basal forebrain and hippocampal volumes would be reduced over time, however more dramatically in females and ApoE ε4 carriers and particularly in the subgroup of individuals with amyloid pathology at baseline. In addition, we wanted to investigate ApoE ε4 genetic variants as a risk determinant for longitudinal basal forebrain and hippocampal atrophy. Our secondary hypothesis was that a greater reduction in basal forebrain and hippocampal volume would be observed over time more dramatically in women and ApoE ε4 homozygotes compared with heterozygotes and, specifically in the subgroup of individuals with amyloid and tau pathology at baseline. Lastly, to complement our analysis, we aimed at examining the effect of sex and Apoe4 genotype on longitudinal measures of executive and working memory functions.

## 2 Methods

### 2.1 Data sources

We obtained data from the Longitudinal Cognitive Impairment and Dementia Study (DELCODE) study, conducted by the German Center for Neurodegenerative Diseases (DZNE) (Jessen et al., 2018). This is an ongoing German multicenter observational study on predementia AD that was initiated in 2014. It aims to characterize early disease stages, in particular SCD, improve prognostics of disease progression and identify new markers for preclinical AD.^1^

All participants or their representatives gave written informed consent. The study protocol was approved by the local institutional review boards and ethics committees of the participating centers. The study was conducted in accordance with the Declaration of Helsinki of 1975 and its subsequent amendments.

### 2.2 Participants

**Figure 1:**
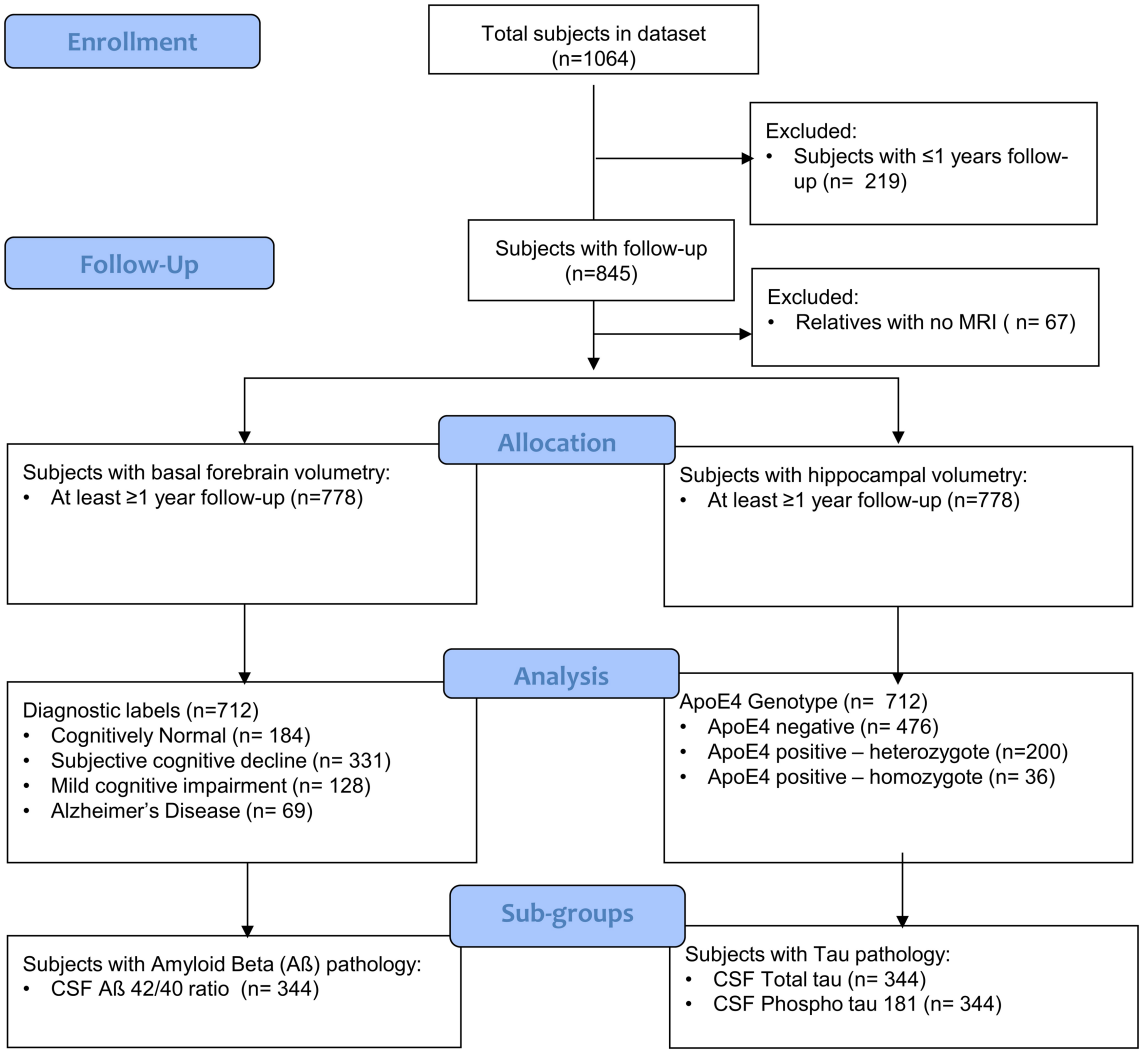
CONSORT flow diagram of the DELCODE study sample. Flow diagram illustrating participant inclusion and exclusion across the DELCODE cohort, stratified by clinical phenotype and *ApoE* ε4 genotype. The diagram details the number of participants assessed for eligibility, excluded (with reasons), and included in the final analyses, including those with available longitudinal MRI data and CSF biomarker measurements. The flow diagram was constructed following the CONSORT template provided by the EQUATOR Network (https://www.equator-network.org).

From the DELCODE cohort, we selected participants with MRI scans available at baseline and at least one follow-up (Figure 1). Diagnosis was extracted at baseline and treated as a time-invariant factor to examine how initial clinical phenotype relates to subsequent longitudinal brain changes. Subjects were grouped according to the following diagnostic groups (Cognitively Normal [CN], Subjective Cognitive Decline [SCD], Mild Cognitive Impairment [MCI], and Alzheimer’s Disease [AD]), and patients ‘relatives. The term SCD was defined as a persistent self-perceived cognitive decline in the absence of objective cognitive impairment, as measured by the cognitive test battery Consortium to Establish a Registry for Alzheimer’s Disease (CERAD). This self-perceived cognitive decline was required to be lasting at least 6 months and be unrelated to an acute event (Jessen et al., 2014). The MCI patients were diagnosed according to the core clinical criteria for MCI of the National Institute on Aging-Alzheimer’s Association (NIA-AA) workgroup guidelines (Albert et al., 2011). The CN participants exhibited no objective cognitive impairment in cognitive tests, no history of neurological or psychiatric disease, and no self-reported cognitive decline. The term “relatives” was defined as individuals who were cognitively normal and had at least one individual with confirmed AD in their immediate family. The DELCODE participants are subject to annual follow-ups, during which clinical, neuropsychological, and imaging assessments are conducted. The sample size was not based on *a priori* power calculation but rather on the current data availability.

### 2.3 Neuropsychological assessment

In this study, we extracted some tests from the CERAD battery which is carried out as part of the DELCODE routine. We used the Mini-Mental State Examination (MMSE), the Digit Span Test total score (forward and backward) from the Wechsler Memory Scale-Revised (WMS-R) to evaluate working memory. Furthermore, we employed the ratio of the Trail Making Test B to A (TMTB/A) as a second measure of executive function.

### 2.4 Bio-material acquisition

Cerebrospinal fluid (CSF) was obtained by means of lumbar puncture following internal consensus recommendations (Jessen et al., 2018). Biomaterial sampling procedures and biomarker measurements for DELCODE have been described elsewhere (Jessen et al., 2018). Amyloid positivity was determined using CSF Aß42/Aß40 ratio and tau pathology by using total tau and phospho tau 181 levels. The cut-off for abnormal concentrations of Aß42/Aß40 ratio was derived from the literature, which applied the widely used threshold of 0.1, commonly employed as a biomarker criterion for Alzheimer’s disease (Janelidze et al., 2016). In contrast, cut-offs for tau biomarkers followed a univariate two-component Gaussian mixture modeling (Colin et al., 2023; Quattrini et al., 2023) fitted to the full set of available baseline values, without sub-group stratification with the midpoint between the means used as a threshold. For total tau, the resulting cutoff was 580 pg./mL, and for phosphorylated tau 181, the cutoff was 76 pg./mL (Supplementary materials, Section1.5–1.6). To control for coherency, we also performed a sensitivity analysis, using the cut-offs predefined in the original DELCODE paper. After grouping individuals based on these cut-offs, we binary coded tau and Aß variables into positive and negative groups.

### 2.5 MRI data acquisition

The MRI data were acquired from nine Siemens 3.0 Tesla MRI scanners (four Verio, one Skyra, three TimTrio, and one Prisma system) using identical acquisition parameters and instructions (Jessen et al., 2018). This is a highly standardized, centrally controlled protocol with harmonized sequences, centralized training, traveling-head qualification, and ACR phantom monitoring across all nine Siemens sites (Jessen et al., 2018). Additionally, to ensure the consistency of image quality throughout the acquisition phase, all scans were subjected to a semi-automated quality check during the study’s conduction. This protocol deviation reporting mechanism enabled the communication of any issues to the respective study sites, facilitating the adjustment of the acquisition at the site. Such procedures of first-level quality checks (e.g., motion artifacts correction) were performed centrally by DZNE Bonn and Magdeburg. After we acquired the data, we performed again a data quality check with default parameters in CAT12 combined with visual inspection (FL), resulting in a total MRI drop-out rate relative to the full acquired dataset of 0.26%. High-resolution structural images were obtained using a sagittal T1-weighted magnetization-prepared rapid gradient echo (MPRAGE) sequence (field of view = 256 × 256 mm^2^; matrix = 256 × 256; isotropic voxel size = 1 mm^3^; repetition time = 2,500 ms; echo time = 4.37 ms; flip angle = 7°; 192 slices; parallel imaging acceleration factor = 2).

### 2.6 MRI data pre-processing and volumetric analysis

We used distinct preprocessing pipelines for analyzing baseline and longitudinal MRI data.

#### 2.6.1 Baseline data pre-processing

Baseline T1-weighted images were preprocessed using the Computational Anatomy Toolbox (CAT12, version 12.8; Gaser and Dahnke, 2016) implemented in SPM12 (release 7,771; Wellcome Centre for Human Neuroimaging, London, UK) running in MATLAB R2020a (The MathWorks Inc., Natick, MA). CAT12’s default voxel-based morphometry (VBM) pipeline was applied (Gaser et al., 2024). Preprocessing steps included bias-field inhomogeneity correction, affine registration to a standard template, and tissue classification into gray matter (GM), white matter (WM), and cerebrospinal fluid (CSF). CAT12 incorporates partial volume estimation and local adaptive segmentation to enhance the delineation of deep gray-matter structures (Gaser et al., 2024). GM and WM tissue maps were spatially normalized to Montreal Neurological Institute (MNI) space using CAT12’s high-dimensional DARTEL/Geodesic Shooting algorithm, followed by modulation to preserve local tissue volumes. Modulated GM maps were smoothed with an 8-mm full-width at half maximum (FWHM) Gaussian kernel.

All images underwent CAT12’s automated quality assurance, including checks for noise, inhomogeneity, and sample homogeneity, complemented by visual inspection. Basal forebrain subregional volumes (anterior-medial and posterior-lateral) were extracted and using a validated cytoarchitectonic and connectivity-based atlas (Fritz et al., 2019; Herdick et al., 2020) and then combined. Bilateral hippocampal GM volumes (left and right hippocampus combined) were extracted from the Harvard–Oxford atlas (Desikan et al., 2006), thresholded at 0.5 GM probability to ensure inclusion of predominantly gray-matter voxels (Tudorascu et al., 2016). Consistent with our previous work and standard procedures in our lab, all atlas-based ROIs were restricted to voxels with gray-matter tissue probability ≥ 0.5 (Grothe et al., 2016; Teipel et al., 2023a,b). Total intracranial volume (TIV) was obtained from CAT12 and used to normalize regional volumes.

#### 2.6.2 Longitudinal data pre-processing

Longitudinal preprocessing was performed separately for subjects with different numbers of MRI sessions, as the number of time points determines the subject-specific reference image and bias correction. We selected follow-up MRI scans that were available in DELCODE. Scans were obtained for delays of approximately 1, 2, 3 or 4 years after the baseline, with an average of 2.8 years follow-up scans. Longitudinal T1-weighted MRI data were preprocessed using the CAT12 (v12.8; Gaser et al., 2024) longitudinal segmentation toolbox implemented in SPM12 (r7771) running in MATLAB R2020a (Ashburner and Ridgway, 2013). For each participant, CAT12 generated a bias-free midpoint image via symmetric diffeomorphic registration across all available time points. Individual scans were realigned to this midpoint, bias-corrected, and segmented into GM, WM, and CSF using CAT12’s partial-volume and local adaptive segmentation algorithms (Gaser et al., 2024). GM and WM maps were normalized to MNI space using CAT12’s high-dimensional DARTEL/Geodesic Shooting framework and modulated to preserve local tissue volumes. The modulated GM maps were smoothed with an 8-mm FWHM Gaussian kernel. Longitudinal quality control followed CAT12’s default procedures, including noise and inhomogeneity metrics, sample homogeneity statistics, and visual inspection (Gaser et al., 2024). The pipeline generated modulated and normalized GM maps for each time point, from which basal forebrain and hippocampal volumes were extracted using atlas-based masks.

### 2.7 Statistical analysis

#### 2.7.1 Demographic characteristics

From an initial sample of 845 subjects with follow-ups, we filtered out relatives (*n* = 67), subjects with less than 1 year follow-up (*n* = 66) and obtained a final sample of 712 (Figure 1). A Wilcoxon rank-sum test was conducted to compare TIV at baseline between male and female participants. Chi-square and ANOVA tests were conducted to examine demographic differences across groups.

#### 2.7.2 Linear mixed effect models

Before entering the brain volumes in the models, we corrected them for TIV using the ratio (proportion) method, whereby regional brain volumes were divided by TIV. This approach represents a conservative (O'Brien et al., 2011) and widely used correction method (Teipel et al., 2014,2024b; Li et al., 2025) based on the assumption of a direct proportional relationship between regional and total brain volume. Alternative correction approaches include the residuals method (Pintzka et al., 2015), analysis of covariance (ANCOVA) (Nordenskjöld et al., 2015), and the power-proportion (PSP) method (Liu et al., 2014), among others. Then we specified two linear mixed-effects models to examine the relationships between normalized longitudinal basal forebrain volume changes (model 1) or hippocampal volume changes (model 2) and several predictors over time. The models included sex, which represents the biological sex of the participants; ApoE ε4 status, categorized into three levels (0 for non-carriers, 1 for heterozygotes, and 2 for homozygotes); time, measured as the number of years between baseline and follow-up MRI measurements; age, the participant’s age at baseline; and baseline diagnosis, which was coded as a categorical variable (0 = cognitively normal, 1 = subjective cognitive decline [SCD], 2 = mild cognitive impairment [MCI], 5 = Alzheimer’s disease [AD]). Baseline diagnosis was included as a covariate to control for clinical disease stage, rather than as a primary predictor, and was therefore not further stratified by sex or ApoE ε4 status. The full model specifications are reported in Section 1 of Supplementary materials. To account for potential variations in the effects of the predictors over time, we included interaction terms between time and each of the fixed effects. Annualized percentage change was derived from the fixed-effect time coefficient of the linear mixed-effects models and expressed relative to the mean baseline volume. We additionally ran simplified models excluding higher-order interaction terms to assess whether model complexity influenced the detectability of ApoE and sex effects. Noteworthy mentioning is that, these models were not used to redefine the primary conclusions.

Since we also aimed at examining the relationship between basal forebrain volume changes and executive functions based on sex and Apoe4 predictors, we created other two models, in which the dependent variables were the Trail Making Test Ratio A/B and Digit Span Total score. The fixed effects included the normalized longitudinal basal forebrain volume, sex, Apoe4 status, time between MRI scans (years), age at baseline and baseline diagnosis. These models also included a random effect structure, with random intercepts and random slopes for time at the subject level. For more detailed information on these models’ specifications, please refer to Section 1.5 of Supplementary materials.

We also stratified subjects based on their baseline CSF amyloid and tau pathology in order to examine basal forebrain volume changes over time in these subgroups. Interaction effects involving amyloid status, time, diagnosis, sex, and ApoE ε4 were specified *a priori* within the mixed-effects models and evaluated using individual model-based hypothesis tests. As these terms do not represent multiple post-hoc comparisons but rather distinct pre-specified regression coefficients estimated within a single model, no false discovery rate (FDR) correction was applied. Exact *p*-values and unstandardized effect sizes with 95% confidence intervals are reported for all fixed effects in Supplementary Tables S5–S11. Additionally, we performed an analysis using a basal forebrain mask that delineated basal forebrain nuclei derived from post-mortem MRI and histological data (Kilimann et al., 2014), as well as a sub-group analysis only in the SCD individuals. All statistical analysis and linear mixed-effects models were implemented in R Studio (R version 4.4.2, R Core Team, 2024) using the lmer() function from the lme4 package, with degrees of freedom and *p*-values estimated using the lmerTest package.

### 2.8 *Post-hoc* sensitivity analysis

*Post-hoc*, we conducted a series of sensitivity analysis. To control for multiple testing within our a priori hypotheses, we applied Holm’s correction within pre-specified families of tests. Specifically, sex main effects and sex × time interactions were evaluated using Type III tests across both regions of interest, and ApoE ε4 baseline effects were evaluated using planned contrasts of estimated marginal means comparing heterozygotes versus non-carriers and homozygotes versus heterozygotes in each region. Other terms in the models, including diagnosis, pathology subgroup variables, and higher-order interaction terms, were included as covariates or exploratory effects and were not subjected to additional multiplicity correction, consistent with standard practice in mixed-effects regression. For all fixed effects, we report unstandardized regression coefficients with 95% confidence intervals as effect size estimates, along with exact *p*-values. To assess whether the null effect of amyloid/tau status on baseline hippocampal volume reflects sufficient statistical power, we calculated the minimum detectable effect (MDE).

In addition, we quantified the magnitude of ApoE ε4 –related group differences using standardized effect sizes (Cohen’s *d*). Cohen’s *d* was calculated for planned ApoE ε4 contrasts in basal forebrain and hippocampus using estimated marginal means derived from the mixed-effects models. Effect sizes were computed using the residual standard deviation of each model, with degrees of freedom inherited from the Kenward–Roger approximation. Regarding TIV method’s correction, we also conducted a sensitivity analysis using residual-based TIV correction after regressing out sex for both basal forebrain and hippocampus.

## 3 Results

### 3.1 Demographic data

The chi-square test for sex differences showed that the number of males (n = 349) and females (n = 363) was balanced (Table 1). The results of the Wilcoxon rank-sum test indicated a statistically significant difference in TIV at baseline between male (mean = 1578.691) and female (mean = 1379.152) participants (W = 2,617,555, *p* < 2.2 × 10^−16^). A highly significant difference was observed in the distribution of ApoE ε4 status, indicating a relatively small proportion of homozygous carriers. Among the ApoE ε4 positive both homozygote and heterozygote (n = 236), 119 (50.04%) were women and 117 (49.57%) were men. Participants were divided in the following diagnostic groups: CN (*N* = 184), SCD (*N* = 331), MCI (*N* = 128) and AD (N = 69). The overall median age of the sample was 71.25 years (interquartile range [IQR] = 9.22). The median number of years of education was 14.00 (IQR = 5), suggesting a relatively well-educated cohort. Lastly, the median duration of follow-up across the sample was 2.8 years [IQR] = 1.75. More detailed demographic characteristics and statistical tests are summarized in Table 1. Among the analyzed 712 MRI scans with repeated measures, a subset of 344 individuals had baseline CSF data, specifically 161 individuals were amyloid-positive (Aß 42/40 ratio) and 183 were amyloid-negative. For phosphorylated tau (p-tau181), 75 individuals were classified as positive and 269 as negative at baseline. In terms of total tau (t-tau), 81 individuals were positive and 263 were negative at baseline.

**Table 1:** Demographic characteristics.

A. Demographics and Clinical Measures							
Baseline Diagnosis^ǂ^	**ApoE ε4 Status**	**N ApoE ε4** ^**Ɨ**^	**N (f/m)** ^*^	**Age [y] mean (95% CI)** ^**§**^	**MMSE mean (95% CI)** ^**{**^	**Education [y] mean (95% CI)** ^**||**^	**Follow-up [y] median (95% CI)**
CN	Negative	147	88 / 59	147	69.50 (69, 70)	14.65 (14, 15)	3.43 (3, 4)
	Positive Heterozygote	34	18 / 16	34	68.88 (67, 71)	14.76 (14, 16)	3.53 (3, 4)
	Positive Homozygote	3	2 / 1	3	69.57 (64, 76)	17.33 (10, 25)	3.79 (2, 5)
SCD	Negative	231	113 / 118	231	71.25 (70, 72)	14.68 (14, 15)	3.25 (3, 3)
	Positive Heterozygote	90	36 / 54	90	71.39 (70, 73)	15.23 (15, 16)	3.25 (3, 3)
	Positive Homozygote	10	6 / 4	10	68.64 (64, 73)	14.80 (13, 16)	3.50 (2, 5)
MCI	Negative	75	32 / 43	75	72.80 (71, 74)	14.23 (13, 15)	2.69 (2, 3)
	Positive Heterozygote	45	24 / 21	45	74.26 (73, 76)	14.47 (13, 15)	2.59 (2, 3)
	Positive Homozygote	8	4 / 4	8	70.67 (67, 75)	13.25 (12, 15)	2.57 (1, 4)
AD	Negative	23	11 / 12	23	77.16 (74, 80)	12.74 (12, 14)	1.70 (1, 2)
	Positive Heterozygote	31	21 / 10	31	75.11 (73, 77)	11.87 (11, 13)	1.98 (1, 2)
	Positive Homozygote	15	8 / 7	15	69.95 (67, 72)	14.47 (13, 16)	2.67 (2 , 3)
B. CSF Biomarker Positivity							
Baseline Diagnosis^ǂ^	**ApoE ε4 Status**	**Total tau+ (GMM) n/N (%)**			**p-tau181+ (GMM) n/N (%)**	**Aβ42/40+ n/N (%)**	
CN	Negative	7/63 (11%)			4/63 (6%)	46/63 (73%)	
	Positive Heterozygote	1/14 (7%)			1/14 (7%)	4/14 (29%)	
	Positive Homozygote	1/1 (100%)			0/1 (0%)	0/1 (0%)	
SCD	Negative	10/105 (9%)			12/105 (11%)	75/105 (71%)	
	Positive Heterozygote	7/44 (16%)			7/44 (16%)	12/44 (27%)	
	Positive Homozygote	1/3 (33%)			0/3 (0%)	0/3 (0%)	
MCI	Negative	14/45 (31%)			9/45 (20%)	19/45 (42%)	
	Positive Heterozygote	10/26 (38%)			13/26 (50%)	3/26 (11%)	
	Positive Homozygote	2/2 (100%)			2/2 (100%)	0/2 (0%)	
AD	Negative	9/16 (56%)			9/16 (56%)	1/16 (6%)	
	Positive Heterozygote	13/17 (76%)			12/17 (71%)	1/17 (6%)	
	Positive Homozygote	6/8 (75%)			6/8 (75%)	0/8 (0%)	

The table illustrates the demographic characteristics of the sample based on baseline diagnosis. **(A)** Amyloid and tau–positive cases across groups are reported as the number of positive participants (n) relative to the total sample size (N), with percentages in parentheses**(B)**. CN, cognitively normal, SCD, subjective cognitive disease, MCI, mild cognitive impairment, AD, Alzheimer’s Disease, Aβ42/40 + = positive amyloid-β 42/40 ratio. Footnotes: ^ǂ^ Chi-square test: baseline diagnosis distribution was highly significant (χ2 = 215.58, *p* < 0.0001). ^Ɨ^Chi-square test: ApoE ε4 status distribution deviated significantly from chance expectations (χ2 = 416.15, *p* < 2.2 × 10−16); the chi-square test for ApoE ε4 status across diagnostic groups was also highly significant (χ2 = 80.11, *p* < 0.001), indicating a strongassociation between diagnosis and ApoE ε4 status, with a higher proportion of ApoE ε4 carriers in the MCI and AD groups. * Chi-square test: no significant differences in the overall distribution of sex (χ2 = 0.276, p = 0.599); however, sex distribution differed significantly across diagnostic groups. § One-way ANOVA: significant variability in age across the sample (F(3,706) = 19.00, *p* < 0.0001); age also differed significantly across diagnostic groups (F(3,706) = 19.26, *p* < 0.0001). {One-way ANOVA: significant variability in MMSE scores (F(3,697) = 418.92, *p* < 0.0001), with AD participants showing significantly lower MMSE scores than all other groups (*p* < 0.0001) and MCI participants scoring significantly lower than both CN and SCD (*p* < 0.0001). || One-way ANOVA: significant variability in years of education (F(3,706) = 10.00, *p* < 0.0001); years of education also differed significantly across diagnostic groups (F(3,706) = 9.75, *p* < 0.0001).

### 3.2 Baseline results

#### 3.2.1 Sex and ApoE ε4 status association with baseline basal forebrain volume

**Figure 2:**
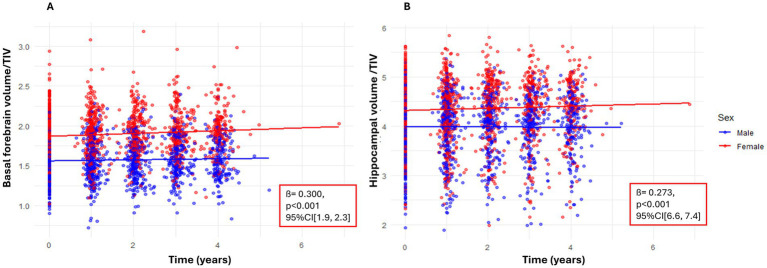
Relative brain volumes predicted by sex. Scatterplot of individual data points in relative basal forebrain **(A)** and hippocampal **(B)** volumes divided by baseline TIV (*y*-axis) over time (years between MRIs) for men (blue) and women (red). *Y*-axis values represent regional volume normalized by TIV. Scales differ across regions and are not directly comparable. Solid lines represent group means for each sex.

**Figure 3:**
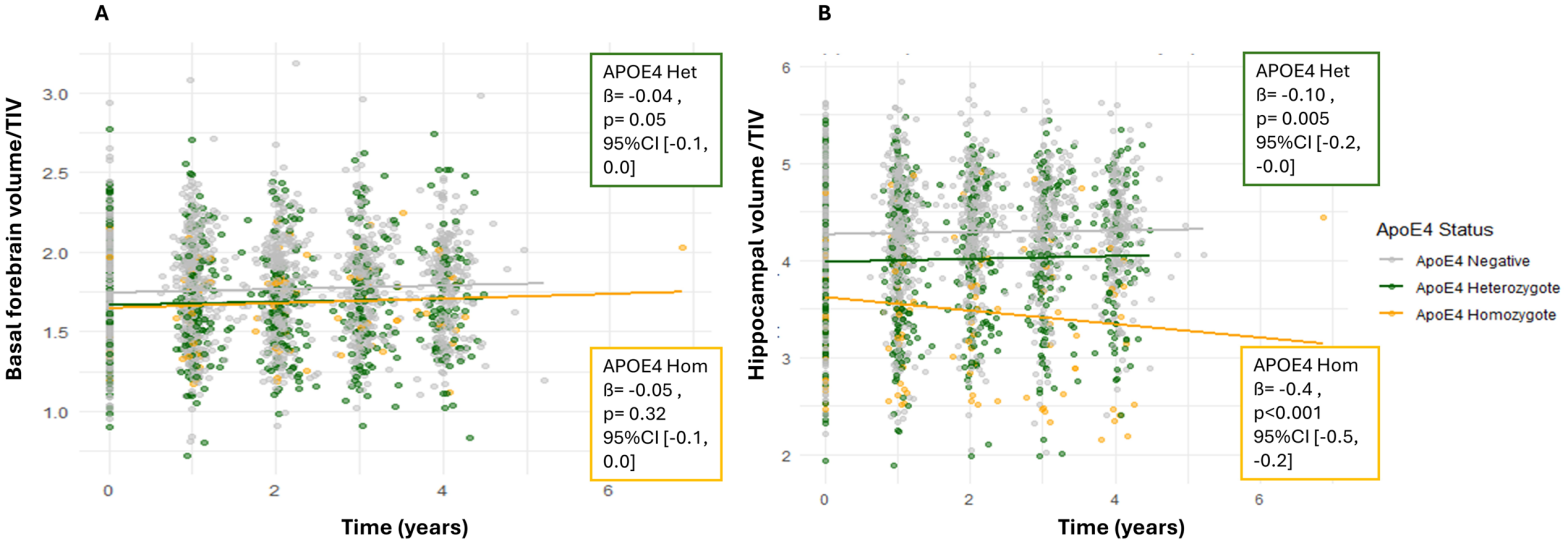
Relative brain volumes predicted by ApoE ε4 status. Scatterplot of individual data points in relative basal forebrain **(A)** and hippocampal **(B)** volumes divided by baseline TIV (*y*-axis) over time (years between MRIs) for ApoE4 negative (gray) ApoE4 heterozygotes (green), and ApoE4 homozygotes (yellow). *Y*-axis values represent regional volume normalized by TIV. Scales differ across regions and are not directly comparable. Solid lines represent group means for each ApoE4 status.

Sex was significantly associated with basal forebrain volume (Table 2), with women having larger basal forebrain volumes than men at baseline (Figure 2A). ApoE ε4 status also showed a significant, but small effect, with only ApoE ε4 heterozygotes exhibiting lower basal forebrain volume at baseline compared to non-carriers (Table 2). These effects remained significant after Holm correction (Table 3). The model showed good fit (AIC = −3006.1; BIC = −2894.3). When directly comparing ApoE ε4 positive heterozygotes and homozygotes, we did not observe a significant difference between the groups (estimate = −0.01, SE = 0.05, t = 0.12, *p* = 0.91, 95% CI [−0.09, 0.08]) (Figure 3A). For all fixed effects and interactions of the basal forebrain model, see Table 2 and Supplementary Table S1. We also carried out a sensitivity analysis using simplified and separate models to look at the effects of sex and ApoE ε4 positivity and obtained concordant results, except that effects of Apoe4 were more visible, especially in homozygotes (Supplementary Table S2). In the post-hoc analysis using non–TIV-adjusted basal forebrain volume, sex was a significant predictor of basal forebrain baseline differences, with female participants showing lower volumes compared to males (estimate = −244.08, t = −13.05, *p* < 0.001, 95% CI [−281.00, −207.00]) (Supplementary Table S3).

**Table 2:** Fixed effects predicting longitudinal basal forebrain and hippocampal volume.

Predictor	Estimate	SE	t	*p*	95% CI [Lower, Upper]
Basal forebrain					
(Intercept)	2.110	0.110	18.41	<0.001	[1.890, 2.340]
Female (vs. male)	0.300	0.020	15.98	<0.001	[0.260, 0.330]
Time (years)	0.040	0.020	2.02	0.044	[0.000, 0.080]
ApoE ε4 heterozygote	−0.040	0.020	−1.97	0.050	[−0.080, 0.000]
ApoE ε4 homozygote	−0.050	0.040	−1.00	0.316	[−0.130, 0.040]
SCD (vs. CN)	−0.040	0.020	−1.71	0.087	[−0.080, 0.010]
MCI (vs. CN)	−0.100	0.030	−3.55	<0.001	[−0.160, −0.050]
AD (vs. CN)	−0.180	0.040	−4.72	<0.001	[−0.250, −0.100]
Age at baseline	−0.010	0.000	−4.15	<0.001	[−0.010, 0.000]
Female × Time	0.000	0.000	1.37	0.171	[0.000, 0.010]
Time × ApoE ε4 heterozygote	0.000	0.000	−0.09	0.925	[−0.010, 0.010]
Time × ApoE ε4 homozygote	0.000	0.010	−0.20	0.841	[−0.020, 0.010]
Time × SCD	0.000	0.000	0.09	0.926	[−0.010, 0.010]
Time × MCI	−0.010	0.000	−1.94	0.053	[−0.020, 0.000]
Time × AD	−0.020	0.010	−2.70	0.007	[−0.040, −0.010]
Hippocampus					
(Intercept)	7.027	0.199	35.37	<0.001	[6.638, 7.417]
Female (vs. male)	0.273	0.033	8.37	<0.001	[0.209, 0.337]
Time (years)	−0.014	0.004	−3.57	<0.001	[−0.022, −0.007]
ApoE ε4 heterozygote	−0.105	0.037	−2.84	0.005	[−0.178, −0.033]
ApoE ε4 homozygote	−0.369	0.078	−4.73	<0.001	[−0.522, −0.216]
SCD (vs. CN)	−0.056	0.040	−1.39	0.165	[−0.134, 0.023]
MCI (vs. CN)	−0.401	0.051	−7.82	<0.001	[−0.501, −0.300]
AD (vs. CN)	−0.899	0.066	−13.62	<0.001	[−1.028, −0.769]
Age at baseline	−0.039	0.003	−13.92	<0.001	[−0.044, −0.033]
Female × Time	0.001	0.004	0.23	0.821	[−0.006, 0.008]
Time × ApoE ε4 heterozygote	−0.007	0.004	−1.77	0.078	[−0.016, 0.001]
Time × ApoE ε4 homozygote	−0.037	0.009	−4.07	<0.001	[−0.054, −0.019]
Time × SCD	−0.009	0.004	−2.02	0.044	[−0.017, −0.000]
Time × MCI	−0.048	0.006	−8.33	<0.001	[−0.060, −0.037]
Time × AD	−0.073	0.009	−8.59	<0.001	[−0.090, −0.056]

The table presents fixed-effect estimates from the linear mixed-effects model examining the impact of demographic and biological predictors on longitudinal basal forebrain and hippocampal volume (normalized for TIV). Unstandardized estimates are reported along with standard errors (SE), degrees of freedom (df), t-values, p-values, and 95% confidence intervals. The intercept reflects the estimated mean basal forebrain volume for the reference group. The full primary model showed good fit (AIC = −3006.1; BIC = −2894.3). SE, Standard Error; df, degrees of freedom; t, t value; p, *p*-value; CI, confidence intervals; CN, Cognitively Normal; SCD, Subjective Cognitive Decline; MCI, Mild Cognitive Impairment; AD, Alzheimer’s disease. Significance codes: ***p ≤ 0.001, **p ≤ 0.01, *p ≤ 0.05, *p* ≤ 0.1, (blank) *p* > 0.1.

**Table 3:** Multiple-comparisons corrected tests of sex and ApoE ε4 effects on basal forebrain and hippocampal volumes.

Region	Effect	Contrast	Estimate	SE	95% CI	p (raw)	p (Holm^ǂ^)
Basal forebrain	Sex (baseline)	Female vs. male	—	—	—	<2 × 10^−16^	<2 × 10^−15^
	Sex × Time	Female × Time	—	—	—	0.101	0.304
	ApoE ε4 (baseline)	Heterozygote vs. non-carrier	−0.042	0.017	[−0.077, −0.008]	0.017	0.034
	ApoE ε4 (baseline)	Homozygote vs. heterozygote	−0.005	0.021	[−0.049, 0.039]	0.824	0.824
	ApoE ε4 × Time	Heterozygote vs. non-carrier	0.000	0.000	[−0.010, 0.010]	0.925	—
	ApoE ε4 × Time	Homozygote vs. non-carrier	0.000	0.010	[−0.020, 0.010]	0.841	—
Hippocampus	Sex (baseline)	Female vs. male	—	—	—	3.1 × 10^−16^	1.5 × 10^−15^
	Sex × Time	Female × Time	—	—	—	0.821	0.821
	ApoE ε4 (baseline)	Heterozygote vs. non-carrier	−0.118	0.033	[−0.182, −0.053]	0.0004	0.0013
	ApoE ε4 (baseline)	Homozygote vs. heterozygote	−0.313	0.039	[−0.395, −0.231]	<0.0001	0.000001
	ApoE ε4 × Time	Heterozygote vs. non-carrier	−0.007	0.004	[−0.016, 0.001]	0.078	—
	ApoE ε4 × Time	Homozygote vs. non-carrier	−0.037	0.009	[−0.054, −0.019]	<0.001	—

SE. Standard Error; df, degrees of freedom; t, t value; p, p-value; CI, confidence intervals; CN, Cognitively Normal; SCD, Subjective Cognitive Decline; MCI, Mild Cognitive Impairment; AD, Alzheimer’s disease. Significance codes: ***p ≤ 0.001, **p ≤ 0.01, *p ≤ 0.05, *p* ≤ 0.1, (blank) *p* > 0.1. ^**ǂ**^Multiple-comparisons correction. Holm correction was applied to pre-specified families of primary hypotheses. Sex effects were evaluated using Type III tests of sex main effects and sex × time interactions across basal forebrain and hippocampus. ApoE ε4 baseline effects were evaluated using planned contrasts of estimated marginal means (heterozygotes vs non-carriers; homozygotes vs heterozygotes) in each region. ApoE ε4-related effects on longitudinal change are shown as model interaction estimates and were not subjected to additional multiplicity correction. Dashes indicate statistics not applicable to Type III tests.

**Figure 4:**
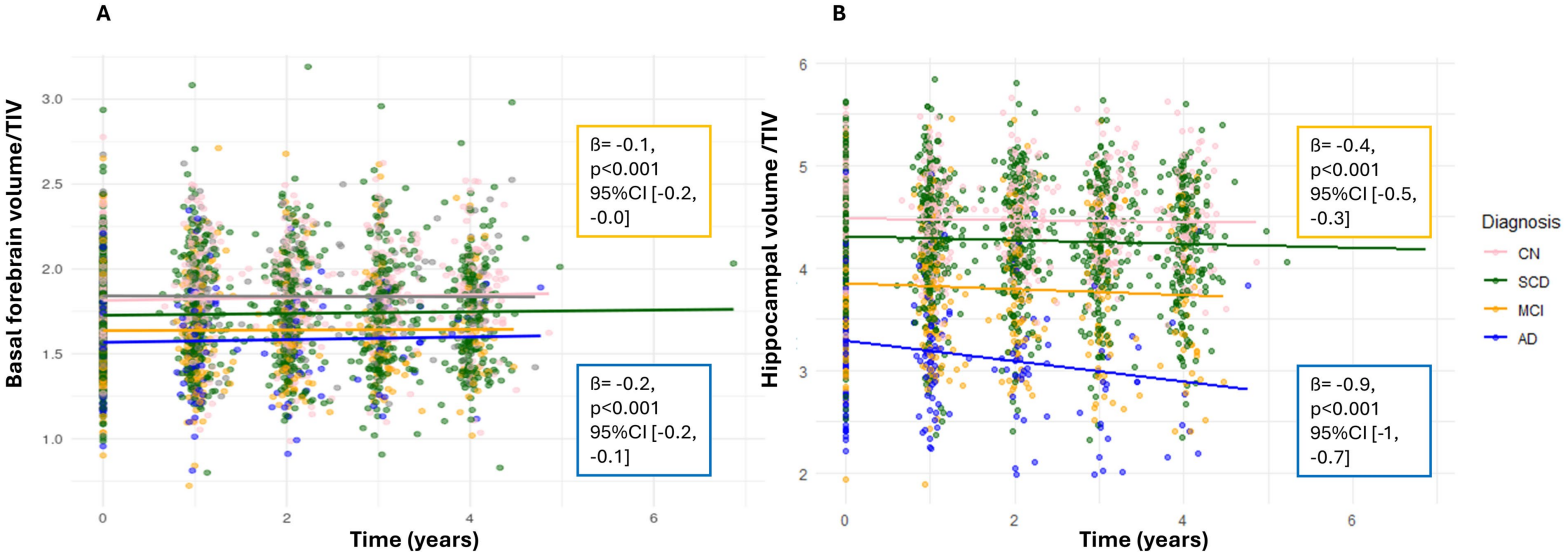
Relative brain volumes predicted by baseline diagnosis. Scatterplot of individual data points in relative basal forebrain **(A)** and hippocampal **(B)** volumes divided by baseline TIV (*y*-axis) over time (years between MRIs) for CN (pink), SCD (green), MCI (yellow), and AD (blue). *Y*-axis values represent regional volume normalized by TIV. Scales differ across regions and are not directly comparable. Solid lines represent group means for each diagnostic label.

**Figure 5:**
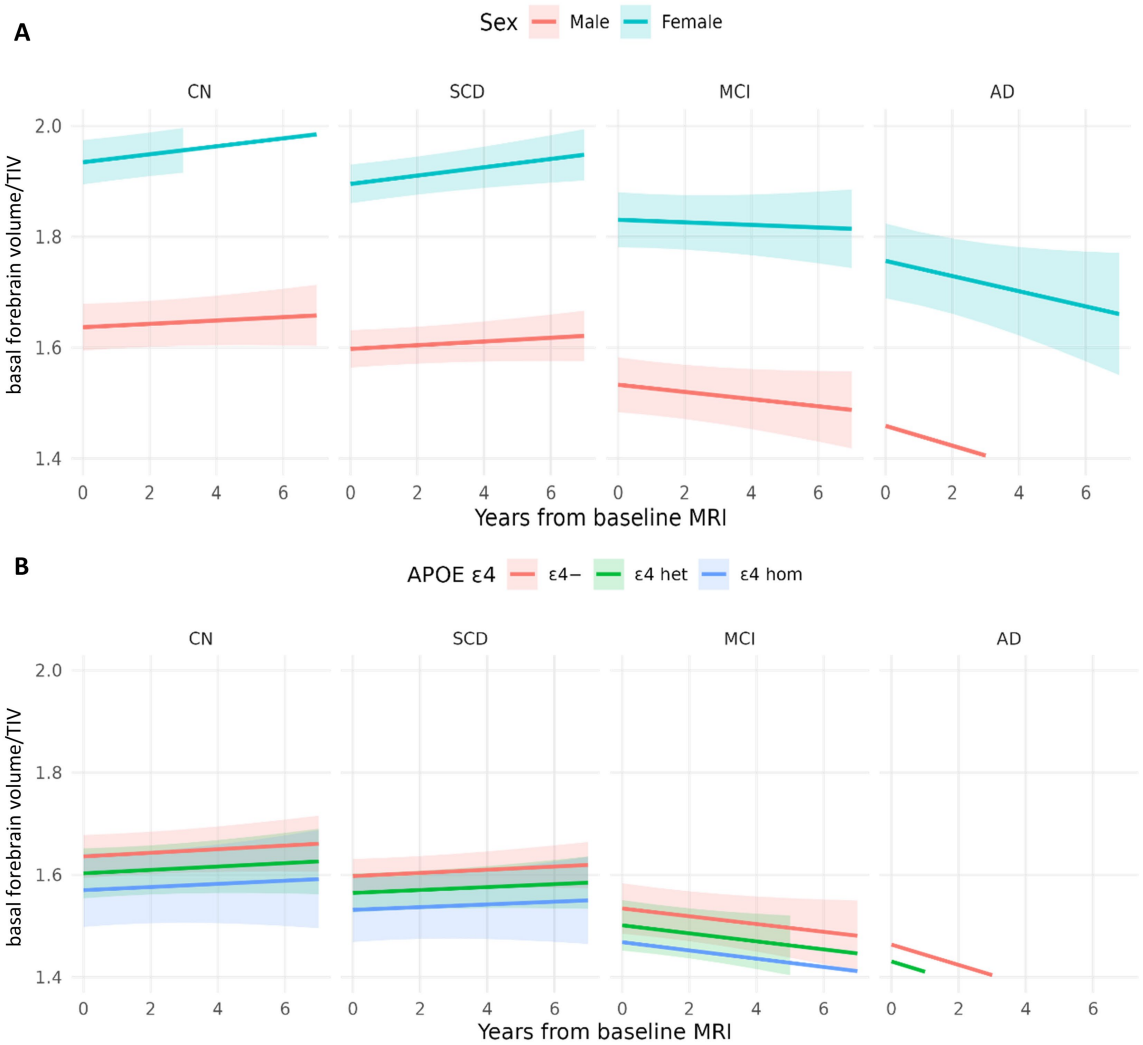
Model-based trajectories of basal forebrain by sex and ApoE ε4. **(A)** Population-level predicted means with 95% CIs at covariate values (age = 71; APOE = 0) and sex-specific intercepts. **(B)** Population-level predicted means with 95% CIs at covariate values (age = 71; sex = male), and APOE4-specific intercepts and slopes.

Older age at baseline was associated with smaller basal forebrain volumes (Table 2). The effect of diagnosis at baseline on volumes in SCD was not significant. Additionally, our sensitivity analysis using the basal forebrain mask from Kilimann et al., 2014 (Supplementary Table S1) showed concordant results, as well as our analysis focusing only on individuals with SCD compared to CN participants, which did not reveal evidence of basal forebrain atrophy predicted by sex or ApoE ε4 positivity (Supplementary Table S4). Indeed, MCI and AD patients showed significantly smaller basal forebrain volumes compared to cognitively normal individuals, with the strongest effect being present in the AD group (Figure 4A). For visualization purposes, population-level predicted means were computed for sex and ApoE ε4 faceted by diagnosis (Figure 5).

Stratification of amyloid negative compared to amyloid positive participants, revealed that higher CSF Aß42/40 ratio, indicative of lower Alzheimer’s disease risk, was significantly associated with larger basal forebrain volume (estimate = 0.20, SE = 0.11, t = 1.91, *p* = 0.05, 95% CI [−0.00, 0.40]) (Supplementary Table S5). Whereas, when participants were stratified for tau pathology (both total tau and p-tau), we found no significant main effect of tau pathology on baseline basal forebrain volume (total tau: estimate = 0.08, SE = 0.11, t = 1.13, *p* = 0.47, 95% CI [−0.12, 0.33]); p-tau: (estimate = 0.11, SE = 0.11, t = 0.94, *p* = 0.35, 95% CI [−0.11, 0.33]) (Supplementary Tables S6, S7).

Basal forebrain volume was not significantly associated with executive performance as measured by the TMT B/A ratio (estimate = −0.10, SE = 0.10, t = −0.78, *p* = 0.43, 95% CI [−0.25, 0.11]), nor did TMT performance differ by sex (estimate = 0.04, SE = 0.10, t = 0.75, *p* = 0.45, 95% CI [−0.07, 0.16]) or ApoE ε4 status (estimate = −0.03, SE = 0.05, t = − 0.61, *p* = 0.54, 95% CI [−0.12, 0.06]). In contrast, greater basal forebrain atrophy was associated with poorer working memory performance on the Digit Span (estimate = − 0.92, SE = 0.34, t = −2.73, *p* = 0.006, 95% CI [−1.60, −0.26]). Older age was associated with worse cognitive performance (estimate = −0.09, SE = 0.02, t = −4.82, *p* < 0.001, 95% CI [−0.12, −0.05]). As expected, participants with MCI and AD showed significantly worse executive function and working memory compared with cognitively normal individuals (TMT B/A: MCI estimate = 0.56, p < 0.001; AD estimate = 0.81, *p* < 0.001; Digit Span: MCI estimate = −1.38, p < 0.001; AD estimate = −3.40, *p* < 0.001), whereas the SCD group did not differ significantly from controls.

#### 3.2.2 Sex and ApoE ε4 status association with baseline hippocampal volume

Sex was significantly associated with hippocampal volume (Table 2), with females having larger hippocampal volumes than males at baseline (Figure 2B). ApoE ε4 status also showed a significant effect, with ApoE ε4 positivity being linked to smaller hippocampal volumes (Figure 3B). In a direct-contrast analysis, we observed that ApoE ε4 heterozygotes had reduced baseline hippocampal volume at baseline compared to non-carriers. Similarly, by comparing ApoE ε4 positive homozygotes and ApoE ε4 negative individuals we could observe a significant difference between the groups, as well as by comparing ApoE ε4 positive heterozygotes and homozygotes. The sensitivity analysis using simplified and separate models to look at the effects of sex and ApoE ε4 positivity led to concordant results (Supplementary Table S8).

Age at baseline was also significantly associated with differences in hippocampal volume, particularly with older individuals having smaller hippocampal volumes (Table 2). The effect of diagnosis at baseline showed a non-significant association with brain volume in SCD. In contrast, MCI and AD patients showed significantly smaller hippocampal volumes compared to cognitively normal individuals, with the strongest effect being present in the AD group (Figure 4B).

Post-hoc analysis did not establish a significant main effect of amyloid pathology on baseline hippocampal volume (estimate = 0.00, SE = 0.18, t = 0.00, *p* = 1, 95% CI [−0.36, 0.36]) (Supplementary Table S9). Furthermore, no substantial interactions of amyloid positivity with sex or with ApoE ε4 were observed (Supplementary Table S9). Similarly, when participants were stratified for tau pathology, no significant main effects were found for both markers on baseline hippocampal volume (total tau: estimate = 0.31, SE = 0.23, t = 1.34, *p* = 0.19, 95% CI [−0.14, 0.77]; phosphotau: estimate = 0.48, SE = 0.26, t = 1.88, *p* = 0.06, 95 %CI [−0.02, 0.98]). Moreover, no significant interaction between tau pathology and sex was found, as well as no significant interaction with ApoE ε4 status (Supplementary Tables S10, S11).

### 3.3 Longitudinal results

#### 3.3.1 Sex and ApoE ε4 status association with longitudinal basal forebrain volume

We observed no significant reduction in basal forebrain volume over time (Table 2, Supplementary Table S1). In linear mixed-effects models, basal forebrain volume showed no significant overall longitudinal change, with an estimated annual change of **+**0.06% per year (95% CI: −0.51 to +0.63%). Despite the absence of a global basal forebrain time effect, diagnosis-by-time interactions indicated accelerated basal forebrain decline in MCI and AD relative to cognitively normal participants. Indeed, having a baseline diagnosis of MCI and AD was found predictive of a significant acceleration in basal forebrain volume loss over time (see Figure 4). Looking at cognition, time between MRIs was associated with higher ratio of TMT B/A (lower executive function), suggesting progressive decline over time (estimate = 0.03, SE = 0.01, t = 2.76, *p* = 0.006, 95% CI [−0.25, 0.11]), whereas when looking at Digit span test as a measure of working memory, time between MRIs did not seem to significantly predict cognitive performance (estimate = 0.03, SE = 0.03, t = 0.88, *p* = 0.38, 95% CI [−0.04, 0.10]).

The post-hoc three-way interaction between CSF Aß42/40 ratio, sex, and time was not significant (Supplementary Table S5). Similarly, the interaction between Aß42/40, time, and ApoE ε4 heterozygosis was not significant. However, the interaction between Aß42/40, time and SCD was significant (estimate = 0.02, SE = 0.01, t = 2.47, *p* = 0.03, 95% CI [0.00, 0.06]). Likewise, a significant interaction was observed between Aß42/40, time, and MCI (estimate = 0.07, SE = 0.01, t = 4.73, *p* < 0.0001, 95% CI [0.04, 0.10]). No significant interaction was found for Aß42/40, time, and AD. Concerning groups stratified per total tau-pathology, we found a non-significant interaction between total tau pathology, sex and time, as well as a non-significant interaction between total tau pathology, ApoE ε4 status and time (Supplementary Table S6). Similarly, we found no significant associations of high p-tau with basal forebrain volume atrophy over time or of p-tau interaction with sex, with ApoE ε4 status and with baseline diagnosis (Supplementary Table S7).

#### 3.3.2 Sex and ApoE ε4 status association with longitudinal hippocampal volume

We found a significant hippocampal volume decline over time (Table 2). Specifically, hippocampal volume exhibited a significant global decline over time, with an estimated annual change of −0.34% per year (95% CI: −0.65 to −0.03%). Additionally, we found a significant interaction between time and ApoE ε4 status, with ApoE ε4 positive homozygotes showing faster hippocampal atrophy over time than heterozygotes (Figure 3). However, we found no significant interaction between sex and time. A significant interaction was found for baseline diagnosis and time, with SCD, MCI and AD patients showing significantly faster hippocampal atrophy, with the strongest decline in AD time. Post-hoc analysis revealed that both baseline CSF Aß42/40 ratio and tau markers (total and p-tau) did not predict hippocampal volume reduction over time (Supplementary Tables S9–S11). Among individuals with MCI, those who were negative to amyloid pathology in the CSF had a significantly slower rate of change in hippocampal volume over time compared to those who were amyloid pathology positive (estimate = 0.05, SE = 0.02, t = 2.65, *p* = 0.01, 95% CI [0.01, 0.81]). For individuals with MCI who were positive for total tau and p-tau, the rate of hippocampal volume decline over time was significantly steeper by −0.051 units per year, compared to cognitively normal tau-negative individuals (Supplementary Tables S10, S11). However, only those with a baseline AD diagnosis who were total tau (not p-tau) positive showed a rate of hippocampal volume decline over time of −0.0539 units per year compared to the same reference group (Supplementary Table S10).

### 3.4 *Post-hoc* analysis

After Holm correction, the main effect of sex remained significant for both basal forebrain and hippocampal volumes. The interaction of sex with time stayed not significant in both region after correction. Regarding ApoE ε4, heterozygous carriers showed significantly lower basal forebrain and hippocampal volumes compared with non-carriers, and these effects survived Holm correction. In the basal forebrain, ApoE ε4 heterozygotes showed moderately lower volume compared with non-carriers (Cohen’s *d* = −0.50, 95% CI [−0.91, −0.09]), whereas the difference between ApoE ε4 homozygotes and heterozygotes was negligible and not statistically meaningful (*d* = −0.06, 95% CI [−0.58, 0.47]). After correction, no longitudinal ApoE ε4 -related differences in basal forebrain atrophy rates were detected. In the hippocampus, ApoE ε4 heterozygotes exhibited substantially lower volume compared with non-carriers, corresponding to a large effect size (*d* = −1.76, 95% CI [−2.72, −0.80]). ApoE ε4 homozygotes showed an even larger reduction in hippocampal volume relative to heterozygotes (*d* = −4.68, 95% CI [−5.91, −3.44]), indicating a strong dependent effect of ApoE ε4 on hippocampal structure. In addition, ApoE ε4 homozygotes exhibited significantly lower hippocampal volume compared with heterozygotes, an effect that also remained significant after correction. All corrected and uncorrected results are summarized in Table 3.

To assess whether the null effect of amyloid/tau status on baseline hippocampal volume reflects sufficient statistical power, we calculated the minimum detectable effect (MDE). Based on the observed standard error (SE = 0.0285) and degrees of freedom (df = 344), the MDE was 0.056. Thus, the present sample was only powered to detect effects of moderate magnitude.

The results of the residual-based TIV correction using sex as regressor were highly consistent with the primary analyses (Supplementary Table S12). Specifically, diagnostic group and age effects remained robust, ApoE ε4 status continued to be associated with smaller hippocampal volumes, and critically, no additional sex × time or ApoE ε4 × time effects emerged in the basal forebrain under residual-based correction. In the hippocampus, faster longitudinal atrophy in ApoE ε4 homozygotes remained evident.

## 4 Discussion

In this study, we investigated whether sex and ApoE ε4 genotype could predict the rate of atrophy of the basal forebrain, using hippocampus as a comparison region. In particular, we hypothesized greater reduction of basal forebrain volume in females and homozygote ApoE ε4 carriers.

### 4.1 Baseline results: amyloid pathology, sex-differences and the role of lifestyle risk factors

At baseline, we found several demographic and genetic factors, which showed small but noteworthy associations with basal forebrain structure. Contrary to our hypothesis, women exhibited larger basal forebrain volumes than men after TIV proportion adjustment method, whereas the direction reversed in non TIV-adjusted analyses, reflecting expected sex differences in overall head size. Similarly, ApoE ε4 status demonstrated only a subtle influence on basal forebrain volume, which contradicts our initial assumption. Indeed, ApoE ε4 heterozygotes showed marginally smaller volumes than non-carriers, but no differences emerged between ε4 heterozygotes and homozygotes. Age effects were as expected, with older participants displaying smaller basal forebrain volumes. Diagnostic comparisons indicated that individuals with subjective cognitive decline did not yet show basal forebrain atrophy, consistent with the notion that early, preclinical complaints are not accompanied by detectable cholinergic degeneration. In contrast, participants with MCI and especially AD exhibited substantially reduced basal forebrain volumes. Neuroinflammation biomarkers provided a similar picture, as lower amyloid burden was associated with larger basal forebrain volume, while total tau and p-tau levels were not significantly related to baseline structure. Together, these findings suggest that basal forebrain alterations at baseline remain subtle in preclinical groups and become more pronounced with advancing clinical stage. However, our findings on baseline sex differences favoring larger basal forebrain and hippocampal volumes in women are not consistent with previous studies showing women higher susceptibility to neurodegenerative processes or have a greater pathological burden (Gamache et al., 2020). On the other hand, they are consistent with the existing literature showing greater susceptibility in men (Cavedo et al., 2018; Neu et al., 2017; Snyder et al., 2016). Men generally have higher cardiovascular risk factors, which can elevate the probability of developing vascular dementia, while women with hypertension appear to have a disproportionately higher risk of developing dementia and exhibit poorer cognitive performance compared to normotensive women (Gamache et al., 2020). This highlights the importance of lifestyle a potential influence in cognition affecting brain volumes differently in men and women. Since our analyses did not include cardiovascular measurements, the interpretation of potential underlying biological mechanisms remains speculative. Therefore, this discussion should be considered as hypothesis generating rather than conclusive, given the observational nature of the data. Nonetheless, we cannot exclude the possibility that men in our sample had higher baseline levels of cardiovascular risk factors, which may have negatively affected brain volumes. Previous research has shown that men are more likely to exhibit signs of neurodegeneration, particularly in the hippocampus (Jack et al., 2017), which partly aligns with our results. Another study has shown that sleep deficit is associated with negative emotions and lower basal nucleus of Meynert functional connectivity with the posterior cingulate cortex in men (Li et al., 2023).

The association between lower basal forebrain volume and poorer working memory performance, but not executive function as assessed by the TMT B/A ratio, suggests that cholinergic degeneration may preferentially affect attentional and short-term memory processes. This interpretation is consistent with experimental and clinical evidence highlighting the critical role of basal forebrain cholinergic projections in sustaining working memory and attentional modulation (Sarter et al., 2003; Mesulam, 2013). In contrast, executive functions rely on distributed frontoparietal networks and multiple neurotransmitter systems, which may explain the absence of a direct association with basal forebrain volume alone (Alvarez and Emory, 2006; Lezak et al., 2012). Similarly, despite well-documented sex differences in cognitive aging and AD risk (Cavedo et al., 2018; Gamache et al., 2020), no significant sex-related differences in cognitive performance were observed in this analysis. These findings may indicate that other factors, such as baseline cognitive reserve, education, or lifestyle variables, may play a more substantial role in determining individual cognitive trajectories.

### 4.2 Longitudinal results

Contrary to our initial assumptions, we found that sex as well as ApoE ε4 status were not associated with basal forebrain rate of atrophy over time. However, this was partly true for the hippocampus, where being a carrier of the ApoE ε4 (heterozygote) was associated with greater decline over time. Nonetheless, sex did not appear to be a predictive factor for brain atrophy in either region. Our findings also showed that hippocampus, but not basal forebrain, exhibited significant progressive atrophy over time. In both regions, atrophy was associated with baseline diagnosis. Previous work suggests that basal forebrain degeneration may occur early in the AD continuum but progress relatively slowly, whereas hippocampal atrophy accelerates at later stages and is more readily detectable over short follow-up intervals (Grothe et al., 2012; Schmitz and Spreng, 2016; Mesulam, 2013). This stage-dependent pattern may explain why hippocampal, but not basal forebrain, atrophy was observed longitudinally in the present study. Longitudinal results showed that amyloid-negative individuals with SCD and MCI exhibited significant slower basal forebrain atrophy over time compared to amyloid-positive individuals with SCD and MCI. The fact that basal forebrain atrophy was detected only in preclinical and prodromal stages of AD is in line with previous literature (Bohnen et al., 2018; Chiesa et al., 2019; Xia et al., 2024). Indeed, Daamen and colleagues found reduced basal forebrain volume in amyloid-positive individuals with SCD compared to amyloid-negative individuals with SCD, suggesting that once amyloid pathology is accompanied by cognitive symptoms, cholinergic degeneration becomes detectable (Daamen et al., 2023). Moreover, a recent study showed evidence that lower basal forebrain volume was associated with faster amyloid-beta accumulation but that, amyloid-beta pathology alone, was not associated with faster basal forebrain atrophy (Yoo et al., 2024). This might suggest that amyloid pathology by itself is insufficient to drive basal forebrain atrophy and that measurable decline appears only when amyloid positivity is accompanied by early cognitive symptoms. This interpretation is also consistent with recent longitudinal work in cognitively unimpaired older adults, where individuals progressing from amyloid negative to amyloid positive pathology showed no accelerated volume loss in the basal forebrain or hippocampus compared to controls (Xia et al., 2024).

In our analysis, tau pathology did not predict longitudinal basal forebrain or hippocampal volumes, nor did it interact with sex or ApoE ε4 status. Instead, hippocampal volume declined significantly faster over time in tau-positive individuals with MCI and AD compared to tau-negative cognitively normal individuals. Nonetheless, our findings are in contrast with evidence increasingly supporting the influence of tau on the cholinergic system (Berry and Harrison, 2023). Indeed, a longitudinal study showed tau accumulation as the strongest predictor of basal forebrain atrophy over time (Yoo et al., 2024). Supporting this claim a recent imaging study showed evidence that the basal forebrain cholinergic system degenerates in individuals at risk for AD presenting tau positive markers (Cantero et al., 2020). The observed differences in our results compared to the literature may be attributable to differences in methodology used. Specifically, the latter study utilized amyloid and tau-PET imaging (Cantero et al., 2020), whereas the former was a cross-sectional analysis from the Alzheimer’s Disease Neuroimaging Initiative (ADNI) cohort with a relatively small sample size (Yoo et al., 2024). In the ADNI study, cytoarchitectonic probabilistic maps of the basal forebrain magnocellular compartments derived from 10 subjects (Záborszky et al., 2018) were employed, with extensions mapped to the amygdala, hippocampus, and neocortex (Yoo et al., 2024). These differences suggest that amyloid-related basal forebrain vulnerability may be more detectable in preclinical and early symptomatic stages, whereas tau-related effects may predominate at later disease stages. Consistently with our findings, a recent cross-sectional study analyzing autopsy data showed that, within the AD subtypes, there seem to be different patterns of basal forebrain vulnerability depending on age, ApoE ε4 status and sex (Hanna Al-Shaikh et al., 2020).

Regarding cognition, time was found to be a significant predictor of executive function but not working memory, as evidenced by worsening performance on the TMT over time, while Digit Span scores remained stable. The decline in TMT performance suggests a gradual deterioration of processing speed and cognitive flexibility, functions that are often sensitive to aging and neurodegenerative processes. In contrast, the stability of Digit Span scores may be attributed to practice effects, as repeated exposure to working memory tasks over multiple testing sessions can lead to performance improvements or maintenance, masking underlying cognitive decline (Teipel et al., 2023a,b). This differential pattern of change across cognitive domains underscores the importance of longitudinal assessments in distinguishing true cognitive decline from practice-related improvements.

### 4.3 The impact of ApoE ε4 on metabolism, hormones, and aging

#### 4.3.1 ApoE ε4 empirical findings

Complementary to our hypothesis we found that ApoE ε4 positive individuals exhibited smaller hippocampal and basal forebrain volumes at baseline. However, only in the hippocampus, ApoE ε4 positivity in homozygotes was associated with accelerated atrophy over time. Contrary to our hypothesis, individuals homozygous for the ApoE ε4 allele did not show greater reductions in basal forebrain volume compared to heterozygotes or ApoE ε4 negative individuals. Instead, the most pronounced volume loss associated with ApoE ε4 homozygosity was observed in the hippocampus. This may be attributable to the relatively small number of ApoE ε4 homozygous individuals in our sample, combined with the limited size and anatomical variability of the basal forebrain region. Interestingly, we also found that older age was associated with smaller basal forebrain and hippocampal volumes. Nonetheless, one limitation of our study is related to the small number of ApoE ε4 homozygous individuals in the sample.

#### 4.3.2 Mechanistic interpretations: age-related, hormonal and metabolic mechanisms

Our findings on ApoE ε4 influence on hippocampal atrophy are in line with the current literature on hippocampus (Gamache et al., 2020). Age as a atrophy predictor is also in line with previous studies on brain age, showing effects on brain volumes (Teipel et al., 2024c). Sex differences in basal forebrain and hippocampal volumes may reflect complex biological mechanisms, including differential hormonal, vascular, and metabolic influences. Prior work has suggested that males may exhibit increased vulnerability to cholinergic and neurodegenerative processes in certain contexts, potentially mediated by cardiovascular risk factors (Gamache et al., 2020). Another line of research suggests that hormonal factors may differentially influence susceptibility to neurodegenerative processes (Moutinho, 2025; Panizzon et al., 2010). Increasing evidence suggests that the ApoE ε4 allele may amplify the effects of hormonal fluctuations and estrogen loss during peri- and post-menopause, potentially contributing to the heightened vulnerability observed in women (Gamache et al., 2020). Specifically, ApoE ε4 has been shown to counteract the neuroprotective effects of estrogen on neurite extension, thereby exacerbating neuronal dysfunction and degeneration (Gamache et al., 2020). Moreover, ApoE ε4 has been implicated in exacerbating glucose hypometabolism, a key feature of early AD pathology (Gamache et al., 2020). However, as the present cohort is largely post-menopausal/post-andropausal and no hormone measurements were available, such mechanisms must be interpreted cautiously. Prior work indicates that hormone-related effects are complex and modulated by ApoE genotype, timing, and treatment characteristics (Mills et al., 2023), underscoring the need for future studies with direct endocrine assessments.

### 4.4 Limitations and future directions

This study has some limitations. First, the basal forebrain is a small and anatomically complex region, which presents challenges for reliable volumetric measurement using standard MRI segmentation techniques. We relied on a basal forebrain mask that was anatomically defined from stereotactic atlases and functionally validated in independent samples (Fritz et al., 2019). Although it is possible that our basal forebrain masks (Fritz et al., 2019) lacked sufficient sensitivity to detect subtle preclinical changes, we conducted a sensitivity analysis using masks that delineate basal forebrain nuclei derived from post-mortem MRI and histological data (Kilimann et al., 2014). This approach yielded comparable results, suggesting that mask resolution was not a major limiting factor in our study. Nevertheless, differences from previously published results may reflect methodological variations (e.g., parcellation approaches, preprocessing pipelines) or cohort characteristics rather than inaccuracies in the applied mask. Second, CSF biomarkers and MRI-derived volumetric measures may not fully capture the extent or distribution of amyloid and tau pathology, nor do they necessarily reflect the functional integrity of the basal forebrain, particularly its cholinergic projections. More sensitive modalities, such as PET imaging capable of detecting cholinergic synaptic dysfunction or diffusion MRI for assessing microstructural changes in cholinergic white matter tracts, may offer a more accurate assessment of basal forebrain degeneration *in vivo*. Future studies employing such modalities could provide a more nuanced understanding of BF involvement in early neurodegeneration. Third, the presence of MCI within the diagnostic spectrum may have obscured early, more subtle changes in basal forebrain structure. MCI is associated with widespread brain alterations, potentially masking region-specific effects of early degeneration. However, our sensitivity analysis focusing on individuals with SCD compared to CN participants did not reveal evidence of basal forebrain atrophy, suggesting that basal forebrain degeneration may occur later in the disease trajectory or may be detectable only through more sensitive imaging techniques.

Regarding sex-based differences, we did not analyze cardiovascular risk factors, and hormonal data were not available. These mechanisms remain speculative and should be examined in future analyses incorporating vascular risk measures. Moreover, TIV represents a major confounding factor when estimating local volumes of interest, as it has been shown that different TIV-adjustment methods can reduce the number of sex differences or generate larger adjusted volumes in females, promoting sex differences that are mainly attributable to TIV variation (Sanchis-Segura et al., 2019). Additionally, we performed a sensitivity analysis using TIV-residuals correction and found concordant results. Thus, our main conclusions did not seem to be dependent on the choice of TIV correction method. Nonetheless, our baseline sex differences following TIV correction should be interpreted as relative, method-dependent effects rather than absolute volumetric differences. A further constraint pertains to the comparatively modest estimate magnitude exhibited by ApoE ε4 copies. This characteristic renders the distinctions between genetic variants with a concomitantly low level of robustness. A larger sample of ApoE ε4 homozygous individuals, which is in turn extremely rare, would be necessary to determine whether the trajectory of neurodegeneration differs significantly from that of heterozygotes, homozygotes and non-carriers, or to observe its interaction with sex. Future studies should also continue to explore whether sex-dependent neurobiological mechanisms, including hormonal influences, interact with genetic risk factors such as ApoE ε4 to shape patterns of brain atrophy.

To conclude, our study revealed that baseline diagnosis, but not sex or ApoE ε4 status, predicted basal forebrain atrophy rate. In contrast, hippocampal volume reduction was predicted by ApoE ε4 homozygosis at baseline and showed significant progressive atrophy over time, particularly in individuals with MCI and AD. Tau and amyloid pathology alone were not predictive of basal forebrain atrophy unless clinical symptoms were present, with amyloid, but not tau, already emerging as a sensitive marker of early cholinergic basal forebrain vulnerability. These findings suggest that basal forebrain degeneration may occur independently of early tau pathology, emphasizing the need for interventions that protect cholinergic function across different genetic and sex groups.

## Data Availability

The data analyzed in this study is subject to the following licenses/restrictions: clinical data. Requests to access these datasets should be directed to projektmanagement.kf@dzne.de.

## Footnotes

1
https://www.dzne.de/forschung/studien/klinische-studien/delcode/
