# Supplementary material for: Predicting longitudinal basal forebrain volume in the Alzheimer’s disease spectrum: the role of sex and ApoE epsilon 4 genotype
Source: Front Neurosci. 2026 Feb 4;20:1730947. doi: 10.3389/fnins.2026.1730947 (PMC12913565; doi:10.3389/fnins.2026.1730947)
Supplement: Supplementary file 1 [file Data_Sheet_1.docx]

Supplementary Material

# **Section 1: Linear Mixed-Effects Models Specification**

## **1.1 Model with normalized longitudinal basal forebrain volume (TIV adjusted)**

We fitted a linear mixed-effects model using the lmer function from the lmerTest package in R to assess the longitudinal trajectory of normalized basal forebrain volume (nlong_BF). We first merged measures of basal forebrain (BF) anterior-medial and posterior-lateral subdivisions into one measure. Then, we adjusted basal forebrain by subtracting baseline total intracranial volume (TIV). Fixed effects included sex (2=females, 1=males), APOE ε4 carrier status (0=Apoe4 negative, 1=Apoe4 heterozygote, 2=Apoe4 homozygote), baseline diagnostic group (0=cognitively normal, 1=SCD, 2=MCI, 5=AD, 100=relative), baseline age (years, continues variable), time between MRIs (years, continues variable), and all two-way interactions between time and sex, APOE status, and diagnosis. Random intercepts and slopes for time were included for each participant to account for within-subject variability in both baseline and longitudinal trajectory.

The model was specified as follows:

**nlongBF∼sex×time + APOE×time + diagnosis×time + sex + APOE + baselinediagnosis + age +**

**(1+time∣subject)**

Post-hoc we conducted a series of analysis. First, we examined basal forebrain volume using anatomically defined masks derived from post-mortem MRI and histological data, as described by Kilimann et al. (2014) (Kilimann et al. 2014). These masks delineate basal forebrain nuclei with high anatomical precision, allowing us to validate the robustness of our findings using an independent and histologically informed parcellation approach.

Second, in order to account for potential sex differences without the influence of total intracranial volume (TIV) adjustment, like observed in (Sanchis-Segura et al. 2019; Sanchis-Segura et al. 2020), we conducted a sensitivity analysis using Model 1 without correcting the basal forebrain volume for TIV. This approach allowed us to examine whether our findings were robust to the exclusion of TIV, which can itself be influenced by sex and may obscure meaningful volumetric differences. Assessing the unadjusted volumes provides additional insight into potential biological differences that might be masked by normalization procedures. For further details, please refer to the supplementary materials, specifically Table S7.

## **1.2 Model with normalized hippocampal volume (TIV adjusted)**

We fitted a linear mixed-effects model using the lmer function from the lmerTest package in R to assess the longitudinal trajectory of normalized basal forebrain volume (nlong_BF). We first merged right and left hippocampus into one measure of bilateral hippocampal volume. Then, we adjusted hippocampal volume by subtracting baseline total intracranial volume (TIV). Fixed effects included sex (2=females, 1=males), APOE ε4 carrier status (0=Apoe4 negative, 1=Apoe4 heterozygote, 2=Apoe4 homozygote), baseline diagnostic group (0=cognitively normal, 1=SCD, 2=MCI, 5=AD, 100=relative), baseline age (years, continues variable), time between MRIs (years, continues variable), and all two-way interactions between time and sex, APOE status, and diagnosis. Random intercepts and slopes for time were included for each participant to account for within-subject variability in both baseline and longitudinal trajectory.

The model was specified as follows:

**nlongHipp∼sex×time + APOE×time + diagnosis×time + sex + APOE + baselinediagnosis + age +**

**(1+time∣subject)**

## **1.3 Model with normalized longitudinal basal forebrain volume (TIV adjusted) stratified by Abeta 42/40 Ratio**

This linear mixed-effects model was constructed to investigate whether baseline cerebrospinal fluid (CSF) amyloid-β42/40 ratio moderates the association between sex, APOE ε4 status, and baseline diagnosis with longitudinal change in basal forebrain volume. The outcome variable was longitudinally adjusted basal forebrain volume, modeled as a function of fixed effects for CSF amyloid-β42/40 ratio (binarized; cut-off:0.1, coded: <0.1=positive “0”, ≥0.1=negative “1”), sex (coded 1 = male, 2 = female), APOE ε4 carrier status (coded 0 = non-carrier, 1 = heterozygote, 2 = homozygote), baseline diagnostic group (coded 0 = cognitively normal, 1 = subjective cognitive decline [SCD], 2 = mild cognitive impairment [MCI], 5 = Alzheimer’s disease [AD]), baseline age (continuous), and time between MRI assessments (years, continuous). The model specifically included three-way interaction terms between amyloid status, time, and each of the following variables: sex, APOE ε4 status, and diagnosis. This allowed for testing whether the effect of sex, APOE genotype, or diagnostic group on basal forebrain atrophy over time was moderated by amyloid positivity.

The model was specified as follows:

**nlongBF ~ Abeta42/40 ratio*(sex * time + APOE * time + diagnosis * time) + sex + APOE + diagnosis + age +**

**(1 + time ∣ participant)**

To account for within-subject variability in both baseline basal forebrain volume and its rate of change over time, the model included a random intercept and random slope for time at the participant level. Participants identified as relatives (prmdiag == 100) were excluded from the analytic sample. The model was estimated using maximum likelihood estimation (REML = FALSE), appropriate for subsequent model comparisons. This analytic approach allows for assessing whether amyloid positivity differentially influences longitudinal basal forebrain atrophy as a function of sex, APOE genotype, and baseline diagnosis.

## **1.4 Model with normalized longitudinal basal forebrain volume (TIV adjusted) stratified by total tau and phospho tau**

We created two linear mixed-effects models to investigate whether baseline CSF tau pathology, measured as total tau and phospho tau (a more specific marker for AD), moderates longitudinal degeneration in the basal forebrain. A binary classification of tau abnormality was derived using Gaussian mixture modeling (GMM). Specifically, GMM with two components (G = 2) was fitted separately to the distributions of total tau and phosphorylated tau 181 using the Mclust algorithm. This unsupervised clustering approach probabilistically assigned individuals to one of two latent subgroups based on the distributional characteristics of their tau levels.

For each biomarker, the means of the two GMM-derived clusters were extracted, and the cutoff value for binary classification was defined as the arithmetic midpoint between these two means. For total tau, the resulting cutoff was 580 pg/mL, and for phosphorylated tau 181, the cutoff was 76 pg/mL. Individuals with values above the respective cutoffs were classified as tau-positive (coded as 1), whereas those with values equal to or below the cutoff were classified as tau-negative (coded as 0). These binary variables were then used as predictors in our mixed-effects models.

The mixed-effects models examined longitudinal change in normalized basal forebrain volume as a function of baseline total tau pathology status. Fixed effects included binary total tau pathology or phosphor tau, sex (coded 1 = male, 2 = female), APOE ε4 status (coded 0 = non-carrier, 1 = heterozygote, 2 = homozygote), baseline diagnosis (0 = cognitively normal, 1 = subjective cognitive decline [SCD], 2 = mild cognitive impairment [MCI], 5 = Alzheimer’s disease [AD]), age at baseline ( continuous), and time between MRI scans (years, continuous).

To test whether the effects of tau pathology on basal forebrain atrophy trajectories were further moderated by demographic and clinical factors, the model included interaction terms between total tau pathology and time as a function of sex, APOE genotype, and diagnosis. Specifically, three-way interaction terms were modeled as: tau * sex * time, total_tau * APOE * time, and total_tau * diagnosis * time. Additional main effects for sex, APOE status, and diagnosis were also included.

A random intercept and random slope for time were specified at the subject level to account for individual differences in both initial volume and rate of change. Participants classified as relatives (prmdiag == 100) were excluded from the analytic sample. The model was estimated using full maximum likelihood (REML = FALSE) to allow for hypothesis testing and model comparison. This modeling framework enabled evaluation of whether CSF total tau status interacts with known Alzheimer's risk factors to influence neurodegeneration within the basal forebrain over time.

The models were specified as follows:

**nlongBF ~ total_tau*(sex * time + APOE * time + diagnosis * time) + sex + APOE + diagnosis + age +**

**(1 + time ∣ participant)**

**nlongBF ~ phospho_tau181*(sex * time + APOE * time + diagnosis * time) + sex + APOE + diagnosis + age +**

**(1 + time ∣ participant)**

## **1.5 Model with longitudinal measures of executive and working memory functions**

We fitted a linear mixed-effects model using the lmer function from the lmerTest package in R to investigate whether longitudinal basal forebrain volume predicts performance on the Trail Making Test B/A ratio (tmtba), a measure of cognitive executive function, and the performance of the Digit Span Test (WMS-R), a measure of working memory. The model(s) included fixed effects for longitudinal basal forebrain volume (nlong_BF), sex (2=females, 1=males), APOE ε4 carrier status (0=Apoe4 negative, 1=Apoe4 heterozygote, 2=Apoe4 homozygote), baseline diagnostic group (0=cognitively normal, 1=SCD, 2=MCI, 5=AD, 100=relative), baseline age (years, continues variable), time between MRIs (years, continues variable). Random intercepts and slopes for time were included for each participant to account for within-subject variability in both baseline performance and longitudinal trajectory.

The model was specified as follows:

**tmtba∼ nlongBF + sex + APOE + time + age + diagnosis +**

**(1 + time ∣ participant)**

**wmstot∼ nlongBF + sex + APOE + time + age + diagnosis +**

**(1 + time ∣ participant)**

# **Section 2: Sensitivity analysis results**

### **Table S1.** **Longitudinal adjusted basal forebrain volumes predicted by sex and apoe4 (Kilimann et al., 2014 basal forebrain’s mask).**

| **Predictor** | **Estimate** | **Std. Error** | **t value** | **p-value** | **95% CI** |
| --- | --- | --- | --- | --- | --- |
| **(Intercept)** | 0.44 | 0.03 | 15.38 | <0.0001 | [0.39, 0.50] |
| **Sex** | 0.07 | 0.01 | 15.83 | <0.0001 | [0.06, 0.08] |
| **Time** | -0.00 | 0.00 | -0.17 | 0.865 | [-0.00, 0.00] |
| **APOE4 Het** | -0.01 | 0.01 | -2.08 | 0.038 * | [-0.02, -0.00] |
| **APOE4 Hom** | -0.01 | 0.01 | -1.29 | 0.196 | [-0.04, 0.01] |
| **SCD** | -0.01 | 0.01 | -1.46 | 0.144 | [-0.02, 0.00] |
| **MCI** | -0.02 | 0.01 | -3.23 | 0.001 ** | [-0.04, -0.01] |
| **AD** | -0.04 | 0.01 | -4.42 | <0.0001 *** | [-0.06, -0.02] |
| **Age** | -0.00 | 0.00 | -4.56 | <0.0001 *** | [-0.00, -0.00] |
| **Sex × Time** | 0.00 | 0.00 | 1.22 | 0.224 | [-0.00, 0.00] |
| **APOE4 Het × Time** | 0.00 | 0.00 | 0.15 | 0.880 | [-0.00, 0.00] |
| **APOE4 Hom × Time** | 0.00 | 0.00 | 0.08 | 0.933 | [-0.00, 0.00] |
| **Time × SCD** | -0.00 | 0.00 | -0.97 | 0.332 | [-0.00, 0.00] |
| **Time × MCI** | -0.00 | 0.00 | -2.48 | 0.013 * | [-0.01, -0.00] |
| **Time × AD** | -0.01 | 0.00 | -2.95 | 0.003 ** | [-0.01, -0.00] |

**Note.** Std.Error= Standard Error, , t=t value, p=p-value, CI= confidence intervals; CN = Cognitively Normal; SCD = Subjective Cognitive Decline; MCI = Mild Cognitive Impairment; AD = Alzheimer’s Disease. ***Significance codes:*** *** p ≤ 0.001, ** p ≤ 0.01, * p ≤ 0.05, . p ≤ 0.1, (blank) p > 0.1.

### **Table S2.** **Longitudinal adjusted basal forebrain volumes: non-linear effects of sex and apoe4 (simplified models).**

| **Predictor** | **Estimate** | **Std. Error** | **t** | **p** | **95% CI**  [Lower, Upper] |
| --- | --- | --- | --- | --- | --- |
| **Sex model** | | | | | |
| (Intercept) | 1.56 | 0.01 | 112.82 | <0.001*** | [1.53, 1.59] |
| **Sex** | 0.31 | 0.02 | 15.98 | <0.001*** | [0.27, 0.35] |
| **Time** | 0.00 | 0.00 | 0.23 | 0.822 | [-0.00, 0.00] |
| **Sex × Time** | 0.01 | 0.00 | 1.70 | 0.090 | [-0.00, 0.01] |
| **Apoe4 model** | | | | | |
| (Intercept) | 1.75 | 0.01 | 127.17 | <0.001 | [1.72, 1.77] |
| **APOE4 Het** vs Neg. | -0.08 | 0.03 | -3.10 | 0.002 | [-0.13, -0.03] |
| **APOE4 Hom** vs Neg. | -0.09 | 0.05 | -1.67 | 0.096 | [-0.19, 0.02] |
| **Time** | 0.00 | 0.00 | 2.23 | 0.026 | [0.00, 0.01] |
| **APOE4 Het × Time** | -0.00 | 0.00 | -0.82 | 0.411 | [-0.01, 0.00] |
| **APOE4 Hom × Time** | -0.01 | 0.01 | -1.27 | 0.204 | [-0.02, 0.00] |

### **Table S3.** **Longitudinal unadjusted basal forebrain volumes predicted by sex and apoe4**

| **Predictor** | **Estimate** | **Std. Error** | ***t* value** | ***p* value** | **95% CI** |
| --- | --- | --- | --- | --- | --- |
| **(Intercept)** | 4028.30 | 116.26 | 34.65 | < 0.001*** | [3800, 4256] |
| **Female** | -244.08 | 18.71 | -13.05 | < 0.001*** | [-281, -207] |
| **Time (years)** | 0.32 | 7.99 | 0.04 | 0.96805 | [-15, 16] |
| **APOE4 Heter** | -26.29 | 21.31 | -1.23 | 0.21783 | [-68, 16] |
| **APOE4 Homo** | -47.29 | 44.83 | -1.06 | 0.29185 | [-135, 41] |
| **SCD** | -24.27 | 22.90 | -1.06 | 0.28941 | [-69, 21] |
| **MCI** | -184.01 | 29.37 | -6.27 | < 0.001*** | [-242, -126] |
| **AD** | -335.43 | 38.03 | -8.82 | <0.001*** | [-410, -261] |
| **Age** | -14.94 | 1.55 | -9.65 | <0.001*** | [-18, -12] |
| **Sex × Time** | 6.40 | 4.37 | 1.47 | 0.14356 | [-2, 15] |
| **Time × APOE Heter** | -0.57 | 5.05 | -0.11 | 0.90978 | [-11, 9.] |
| **Time × APOE Homo** | 1.17 | 10.88 | 0.11 | 0.91418 | [-20, 23] |
| **Time × SCD** | -2.06 | 5.01 | -0.41 | 0.68086 | [-12, 8] |
| **Time × MCI** | -16.88 | 7.03 | -2.40 | 0.01668 * | [-31, -3.10] |
| **Time × AD** | -35.53 | 11.08 | -3.21 | 0.00139 ** | [-57, -14] |

**Note.** Results are uncorrected for TIV=Total Intracranial Volume (Sanchis-Segura et al., 2019;2020)**.** Std.Error= Standard Error, , t=t value, p=p-value, CI= confidence intervals; CN = Cognitively Normal; SCD = Subjective Cognitive Decline; MCI = Mild Cognitive Impairment; AD = Alzheimer’s Disease. ***Significance codes:*** *** p ≤ 0.001, ** p ≤ 0.01, * p ≤ 0.05, . p ≤ 0.1, (blank) p > 0.1.

### **Table S4**. **Sub-group analysis CN vs SCD**.

| **Predictor** | **Estimate** | **Std. Error** | **t value** | **p-value** | **95% CI (Lower, Upper)** |
| --- | --- | --- | --- | --- | --- |
| **(Intercept)** | 2.187 | 0.128 | 13.929 | <0.001 *** | [1.62, 2.15] |
| **Sex** | 0.299 | 0.021 | 13.843 | <0.001 *** | [0.26, 0.34] |
| **Time** | 0.0036 | 0.0033 | -0.398 | 0.287 | [-0.01, 0.01] |
| **APOE4 Het** | -0.0377 | 0.0208 | -1.857 | 0.071 . | [-0.09, 0.00] |
| **APOE4 Hom** | -0.0434 | 0.0671 | -0.647 | 0.5178 | [-0.17, 0.09] |
| **Diagnosis (SCD vs. CN)** | -0.0369 | 0.0223 | -1.651 | 0.098 . | [-0.08, 0.01] |
| **Age** | -0.0077 | 0.0018 | -4.327 | <0.001 *** | [-0.01, -0.00] |
| **Sex × Time** | 0.0053 | 0.0033 | 1.688 | 0.107 | [-0.00, 0.01] |
| **APOE4 Het × Time** | -0.0010 | 0.0032 | 0.199 | 0.752 | [-0.01, 0.01] |
| **APOE4 Hom × Time** | -0.0083 | 0.0099 | -0.832 | 0.406 | [-0.03, 0.01] |
| **SCD × Time** | -0.00035 | 0.0034 | -0.111 | 0.918 | [-0.01, 0.01] |

**Note.** Std.Error= Standard Error, , t=t value, p=p-value, CI= confidence intervals; CN = Cognitively Normal; SCD = Subjective Cognitive Decline; MCI = Mild Cognitive Impairment; AD = Alzheimer’s Disease. ***Significance codes:*** *** p ≤ 0.001, ** p ≤ 0.01, * p ≤ 0.05, . p ≤ 0.1, (blank) p > 0.1.

### **Table S5. Amyloidbeta ratio predicting longitudinal normalized basal forebrain volume.**

| **Fixed Effect** | **Estimate** | **Std. Error** | **t value** | **p-value** | **95% CI (Lower, Upper)** |
| --- | --- | --- | --- | --- | --- |
| (Intercept) | 1.6663 | 0.1949 | 8.549 | < 0.0001*** | [1.28, 2.05] |
| **Aβ42/40 ratio** | 0.1999 | 0.1045 | 1.913 | 0.0566 . | [-0.00, 0.40] |
| **Sex** | 0.3356 | 0.0376 | 8.926 | < 0.0001*** | [0.26, 0.41] |
| **Time** | 0.0037 | 0.0118 | 0.311 | 0.7561 | [-0.02, 0.03] |
| **APOE Het** | -0.0536 | 0.0386 | -1.389 | 0.1657 | [-0.13, 0.02] |
| **APOE Homo** | -0.0224 | 0.0746 | -0.300 | 0.7644 | [-0.17, 0.12] |
| **SCD** | 0.0296 | 0.0566 | 0.523 | 0.6014 | [-0.08, 0.14] |
| **MCI** | -0.0379 | 0.0594 | -0.639 | 0.5232 | [-0.15, 0.08] |
| **AD** | -0.0931 | 0.0660 | -1.410 | 0.1593 | [-0.22, 0.04] |
| **Age at baseline** | -0.0065 | 0.0025 | -2.633 | 0.0088 ** | [-0.01, -0.00] |
| **Sex × Time** | 0.0073 | 0.0063 | 1.156 | 0.2487 | [-0.01, 0.02] |
| **Time × APOE Het** | 0.0014 | 0.0066 | 0.204 | 0.8388 | [-0.01, 0.01] |
| **Time × APOE Homo** | 0.0137 | 0.0113 | 1.213 | 0.2269 | [-0.01, 0.04] |
| **Time x SCD** | -0.0083 | 0.0084 | -0.990 | 0.3232 | [-0.02, 0.01] |
| **Time × MCI** | -0.0389 | 0.0091 | -4.299 | 0.00003*** | [-0.06, -0.02] |
| **Time × AD** | -0.0412 | 0.0117 | -3.513 | 0.00051*** | [-0.06, -0.02] |
| **Aβ42/40 × Sex** | -0.0529 | 0.0553 | -0.955 | 0.3401 | [-0.16, 0.06] |
| **Aβ42/40 × time** | 0.0006 | 0.0163 | 0.037 | 0.9708 | [-0.03, 0.03] |
| **Aβ42/40 × APOE Het** | 0.0412 | 0.0718 | 0.574 | 0.5665 | [-0.10, 0.18] |
| **Aβ42/40 × SCD** | -0.1439 | 0.0721 | -1.996 | 0.0467 * | [-0.29, -0.00] |
| **Aβ42/40 × MCI** | -0.0843 | 0.0876 | -0.962 | 0.3368 | [-0.26, 0.09] |
| **Aβ42/40 × AD** | -0.1270 | 0.1922 | -0.661 | 0.5092 | [-0.50, 0.25] |
| **Aβ42/40 × Sex × time** | -0.0104 | 0.0089 | -1.178 | 0.2401 | [-0.03, 0.01] |
| **Aβ42/40 × APOE Het x time** | -0.0183 | 0.0114 | -1.599 | 0.1111 | [-0.04, 0.00] |
| **Aβ42/40 × SCD × Time** | 0.0230 | 0.0107 | 2.147 | 0.0329 * | [0.00, 0.06] |
| **Aβ42/40 × MCI × Time** | 0.0710 | 0.0150 | 4.732 | 0.0000037 *** | [0.04, 0.10] |
| **Aβ42/40 × AD × Time** | 0.0574 | 0.0320 | 1.794 | 0.0744 . | [-0.01, 0.12] |

**Note.** Std.Error= Standard Error, , t=t value, p=p-value, CI= confidence intervals; CN = Cognitively Normal; SCD = Subjective Cognitive Decline; MCI = Mild Cognitive Impairment; AD = Alzheimer’s Disease. ***Significance codes:*** *** p ≤ 0.001, ** p ≤ 0.01, * p ≤ 0.05, . p ≤ 0.1, (blank) p > 0.1.

### **Table S6. Total tau predicting longitudinal normalized basal forebrain.**

| **Predictor** | **Estimate** | **Std. Error** | **t-value** | **p-value** | **95% CI (Lower, Upper)** |
| --- | --- | --- | --- | --- | --- |
| (Intercept) | 1.827 | 0.179 | 10.32 | <0.001 *** | [1.49, 2.17] |
| **Total tau** | 0.079 | 0.110 | 1.13 | 0.470 | [-0.12, 0.33] |
| **Sex** | 0.334 | 0.031 | 10.89 | <0.001 *** | [0.27, 0.39] |
| **Time** | 0.014 | 0.008 | 1.51 | 0.093 . | [-0.00, 0.03] |
| **APOE4** | -0.041 | 0.031 | -1.19 | 0.188 | [-0.10, 0.02] |
| **Diagnosis** | -0.021 | 0.014 | -1.31 | 0.128 | [-0.05, 0.01] |
| **Age** | -0.008 | 0.002 | -3.37 | 0.0009 *** | [-0.01, -0.00] |
| **Sex * time** | -0.003 | 0.005 | -0.59 | 0.527 | [-0.01, 0.01] |
| **Time * APOE** | -0.005 | 0.005 | -1.40 | 0.285 | [-0.02, 0.00] |
| **Time * diagnosis** | -0.002 | 0.003 | -0.24 | 0.459 | [-0.01, 0.00] |
| **Totaltau * sex** | -0.069 | 0.066 | -1.27 | 0.293 | [-0.20, 0.07] |
| **Totaltau * time** | -0.024 | 0.019 | -0.95 | 0.202 | [-0.07, 0.01] |
| **Totaltau * APOE4** | 0.022 | 0.054 | 0.14 | 0.684 | [-0.09, 0.13] |
| **Totaltau * diagnosis** | -0.016 | 0.022 | -0.52 | 0.474 | [-0.06, 0.03] |
| **Totaltau * sex * time** | 0.017 | 0.012 | 0.84 | 0.159 | [-0.00, 0.04] |
| **Totaltau * time * APOE4** | 0.015 | 0.009 | 2.09 | 0.108 | [-0.00, 0.04] |
| **Totaltau * time * diagnosis** | -0.007 | 0.005 | -2.13 | 0.152 | [-0.02, 0.00] |

**Note.** Std.Error= Standard Error, , t=t value, p=p-value, CI= confidence intervals; CN = Cognitively Normal; SCD = Subjective Cognitive Decline; MCI = Mild Cognitive Impairment; AD = Alzheimer’s Disease. ***Significance codes:*** *** p ≤ 0.001, ** p ≤ 0.01, * p ≤ 0.05, . p ≤ 0.1, (blank) p > 0.1.

### **Table S7.P-tau predicting longitudinal normalized basal forebrain volume**

| **Predictor** | **Estimate** | **Std. Error** | **t value** | **p-value** | **95% CI** |
| --- | --- | --- | --- | --- | --- |
| **(Intercept)** | 1.836 | 0.178 | 10.332 | <0.001 *** | [1.50, 2.18] |
| **Phosphotau181** | 0.106 | 0.113 | 0.938 | 0.349 | [-0.11, 0.33] |
| **Sex** | 0.333 | 0.031 | 10.879 | <0.001 *** | [0.27, 0.39] |
| **Time** | 0.015 | 0.009 | 1.723 | 0.086 . | [-0.00, 0.03] |
| **APOE4** | -0.043 | 0.031 | -1.395 | 0.164 | [-0.10, 0.02] |
| **Diagnosis** | -0.020 | 0.014 | -1.369 | 0.172 | [-0.05, 0.01] |
| **Age** | -0.008 | 0.002 | -3.374 | 0.001 *** | [-0.01, -0.00] |
| **Sex * time** | -0.004 | 0.005 | -0.783 | 0.434 | [-0.01, 0.01] |
| **Time * APOE4** | -0.006 | 0.005 | -1.105 | 0.271 | [-0.02, 0.00] |
| **Time * diagnosis** | -0.002 | 0.003 | -0.793 | 0.428 | [-0.01, 0.00] |
| **Phosphotau * sex** | -0.065 | 0.068 | -0.952 | 0.342 | [-0.20, 0.10] |
| **Phosphotau * time** | -0.029 | 0.020 | -1.480 | 0.140 | [-0.07, 0.01] |
| **Phosphotau * APOE4** | 0.018 | 0.055 | 0.326 | 0.745 | [-0.10, 0.13] |
| **Phosphotau * diagnosis** | -0.016 | 0.022 | -0.717 | 0.474 | [-0.06, 0.03] |
| **Phosphotau * sex * time** | 0.021 | 0.012 | 1.688 | 0.093 . | [-0.00, 0.04] |
| **Phosphotau * time * APOE4** | 0.017 | 0.010 | 1.710 | 0.089 . | [-0.00, 0.04] |
| **Phosphotau * time * diagnosis** | -0.009 | 0.005 | -1.912 | 0.057 . | [-0.02, 0.00] |

**Note.** Std.Error= Standard Error, , t=t value, p=p-value, CI= confidence intervals; CN = Cognitively Normal; SCD = Subjective Cognitive Decline; MCI = Mild Cognitive Impairment; AD = Alzheimer’s Disease. ***Significance codes:*** *** p ≤ 0.001, ** p ≤ 0.01, * p ≤ 0.05, . p ≤ 0.1, (blank) p > 0.1.

### **Table S8.** **Longitudinal adjusted hippocampal volumes: non-linear effects of sex and apoe4 (simplified models).**

| **Predictor** | **Estimate** | **Std. Error** | **t** | **p** | **95% CI [Lower, Upper]** |
| --- | --- | --- | --- | --- | --- |
| **Sex model** | | | | | |
| (Intercept) | 4.01 | 0.03 | 122.69 | <0.001*** | [3.94, 4.07] |
| **Sex** | 0.33 | 0.05 | 7.11 | <0.001*** | [0.24, 0.41] |
| **Time** | -0.04 | 0.00 | -12.71 | <0.001*** | [-0.04, -0.03] |
| **Sex × Time** | 0.00 | 0.00 | 0.23 | 0.817 | [-0.01, 0.01] |
| **Apoe4 model** | | | | | |
| (Intercept) | 4.28 | 0.03 | 153.30 | <0.001*** | [4.23, 4.34] |
| **APOE4 Het** vs Neg. | -0.27 | 0.05 | -5.22 | <0.001*** | [-0.37, -0.17] |
| **APOE4 Hom** vs Neg. | -0.64 | 0.11 | -6.12 | <0.001*** | [-0.85, -0.44] |
| **Time** | -0.03 | 0.00 | -11.90 | <0.001*** | [-0.03, -0.02] |
| **APOE4 Het × Time** | -0.02 | 0.00 | -3.74 | <0.001*** | [-0.03, -0.01] |
| **APOE4 Hom × Time** | -0.06 | 0.01 | -6.81 | <0.001*** | [-0.08, -0.05] |

### **Table S9. Amyloidbeta ratio predicting longitudinal normalized hippocampal volume**.

| **Parameter** | **Estimate** | **Std. Error** | **t-value** | **p-value** | **95% CI** |
| --- | --- | --- | --- | --- | --- |
| (Intercept) | 6.559 | 0.352 | 18.66 | <0.0001*** | [6, 7.25] |
| **Ratio Abeta42/40** | 0.001 | 0.185 | 0.003 | 0.998 | [-0.36, 0.36] |
| **Sex** | 0.302 | 0.066 | 4.55 | <0.0001*** | [0.17, 0.43] |
| **Time** | -0.017 | 0.014 | -1.20 | 0.233 | [-0.04, 0.01] |
| **APOE Het** | -0.133 | 0.068 | -1.96 | 0.051 | [-0.30, 0.00] |
| **APOE Homo** | -0.172 | 0.132 | -1.31 | 0.193 | [-0.43, 0.09] |
| **SCD** | -0.157 | 0.100 | -1.57 | 0.118 | [-0.35, 0.04] |
| **MCI** | -0.494 | 0.105 | -4.71 | <0.0001*** | [-0.70, -0.29] |
| **AD** | -0.903 | 0.117 | -7.75 | <0.0001*** | [-1.13, -0.67] |
| **Age at baseline** | -0.037 | 0.004 | -8.16 | <0.0001*** | [-0.05, -0.03] |
| **Sex × Time** | 0.003 | 0.007 | 0.46 | 0.648 | [-0.01, 0.02] |
| **Time × APOE Het** | -0.011 | 0.008 | -1.37 | 0.171 | [-0.03, 0.00] |
| **Time × APOE Homo** | -0.026 | 0.014 | -1.85 | 0.065 | [-0.05, 0.00] |
| **Time x SCD** | -0.02 | 0.010 | -1.80 | 0.079 | [-0.38, 0.00] |
| **Time × MCI** | -0.070 | 0.011 | -6.47 | <0.0001*** | [-0.10, -0.05] |
| **Time × AD** | -0.081 | 0.013 | -6.11 | <0.0001*** | [-0.11, -0.05] |
| **Aβ42/40 × Sex** | 0.027 | 0.097 | 0.27 | 0.786 | [-0.16, 0.22] |
| **Aβ42/40 × time** | 0.015 | 0.019 | 0.749 | 0.454 | [-0.02, 0.05] |
| **Aβ42/40 × APOE Het** | 0.133 | 0.12 | 1.055 | 0.291 | [-0.11, 0.38] |
| **Aβ42/40 × SCD** | 0.050 | 0.127 | 0.397 | 0.691 | [-0.20,0.30] |
| **Aβ42/40 × MCI** | 0.100 | 0.155 | 0.646 | 0.518 | [-0.20, 0.40] |
| **Aβ42/40 × AD** | 0.352 | 0.339 | 1.040 | 0.299 | [-0.31, 1.02] |
| **Aβ42/40 × Sex × time** | -0.016 | 0.010 | -1.493 | 0.136 | [-0.04, 0.01] |
| **Aβ42/40 × APOE Het x time** | 0.013 | 0.013 | 0.951 | 0.342 | [-0.01, 0.039] |
| **Aβ42/40 × SCD × Time** | 0.024 | 0.013 | 1.829 | 0.068 | [-0.00, 0.05] |
| **Aβ42/40 × MCI × Time** | 0.046 | 0.018 | 2.65 | **0.009**** | [0.01, 0.81] |
| **Aβ42/40 × AD × Time** | 0.053 | 0.038 | 1.367 | **0.172** | [-0.02, 0.13] |

**Note.** Std.Error= Standard Error, , t=t value, p=p-value, CI= confidence intervals; CN = Cognitively Normal; SCD = Subjective Cognitive Decline; MCI = Mild Cognitive Impairment; AD = Alzheimer’s Disease. ***Significance codes:*** *** p ≤ 0.001, ** p ≤ 0.01, * p ≤ 0.05, . p ≤ 0.1, (blank) p > 0.1.

### **Table S10. Total tau predicting longitudinal normalized hippocampal volume.**

| **Parameter** | **Estimate** | **Std.Error** | **t** | **p-value** | **95% CI** |
| --- | --- | --- | --- | --- | --- |
| **(Intercept)** | 6.6323 | 0.3231 | 20.526 | < 2e-16 *** | [6.0002, 7.2645] |
| **Total tau** | 0.3115 | 0.2323 | 1.341 | 0.1807 | [-0.1438, 0.7669] |
| **Sex** | 0.3629 | 0.0545 | 6.664 | 1.06e-10 *** | [0.2562, 0.4696] |
| **Time (Years)** | -0.0092 | 0.0107 | -0.865 | 0.3876 | [-0.0301, 0.0117] |
| **APOE+ Heterozygote** | -0.0831 | 0.0618 | -1.346 | 0.1793 | [-0.2042, 0.0380] |
| **APOE+ Homozygote** | -0.0702 | 0.2300 | -0.305 | 0.7604 | [-0.5210, 0.3806] |
| **SCD** | -0.1191 | 0.0657 | -1.811 | 0.0709 . | [-0.2489, 0.0108] |
| **MCI** | -0.4362 | 0.0840 | -5.193 | 3.56e-07 *** | [-0.6000, -0.2725] |
| **AD** | -0.8341 | 0.1436 | -5.807 | 1.44e-08 *** | [-1.1156, -0.5526] |
| **Age at Baseline** | -0.0386 | 0.0044 | -8.803 | < 2e-16 *** | [-0.0472, -0.0301] |
| **Time** **× Sex** | -0.0060 | 0.0058 | -1.040 | 0.2993 | [-0.0174, 0.0053] |
| **Time** **× APOE+ Heter** | -0.0094 | 0.0067 | -1.398 | 0.1632 | [-0.0224, 0.0037] |
| **Time** **× APOE+ Homo** | -0.0458 | 0.0258 | -1.776 | 0.0769 . | [-0.0963, 0.0047] |
| **SCD × Time** | 0.0009 | 0.0066 | 0.137 | 0.8913 | [-0.0121, 0.0139] |
| **MCI × Time** | -0.0360 | 0.0094 | -3.820 | 0.0002 *** | [-0.0544, -0.0176] |
| **AD × Time** | -0.0366 | 0.0191 | -1.914 | 0.0563 . | [-0.0732, 0.0000] |
| **Tau × Sex** | -0.2199 | 0.1179 | -1.865 | 0.0630 . | [-0.4500, 0.0101] |
| **Tau × Time** | 0.0001 | 0.0248 | 0.002 | 0.9983 | [-0.0486, 0.0487] |
| **Tau × APOE Het** | -0.1754 | 0.1255 | -1.398 | 0.1631 | [-0.4213, 0.0704] |
| **Tau × APOE Hom** | -0.2206 | 0.2796 | -0.789 | 0.4306 | [-0.7684, 0.3272] |
| **Tau × SCD** | -0.0635 | 0.1941 | -0.327 | 0.7437 | [-0.4439, 0.3170] |
| **Tau × MCI** | -0.0393 | 0.1911 | -0.206 | 0.8371 | [-0.4139, 0.3353] |
| **Tau × AD** | 0.0233 | 0.2226 | 0.105 | 0.9168 | [-0.4130, 0.4596] |
| **Tau × Sex × Time** | 0.0027 | 0.0132 | 0.206 | 0.8369 | [-0.0231, 0.0286] |
| **Tau × Time × APOE Het** | 0.0005 | 0.0151 | 0.030 | 0.9758 | [-0.0291, 0.0300] |
| **Tau × Time × APOE Hom** | 0.0341 | 0.0309 | 1.103 | 0.2708 | [-0.0261, 0.0942] |
| **Tau × Time × SCD** | -0.0332 | 0.0204 | -1.624 | 0.1056 | [-0.0732, 0.0069] |
| **Tau × Time × MCI** | -0.0514 | 0.0203 | -2.528 | 0.0120 * | [-0.0912, -0.0115] |
| **Tau × Time × AD** | -0.0539 | 0.0273 | -1.973 | 0.0492 * | [-0.1074, -0.0004] |

**Note.** Std.Error= Standard Error, , t=t value, p=p-value, CI= confidence intervals; CN = Cognitively Normal; SCD = Subjective Cognitive Decline; MCI = Mild Cognitive Impairment; AD = Alzheimer’s Disease. ***Significance codes:*** *** p ≤ 0.001, ** p ≤ 0.01, * p ≤ 0.05, . p ≤ 0.1, (blank) p > 0.1.

### **Table S11. P-tau predicting longitudinal normalized hippocampal volume.**

| **Predictor** | **Estimate** | **Std. Error** | **t** | **p-value** | **95% CI** |
| --- | --- | --- | --- | --- | --- |
| **(Intercept)** | 6.643 | 0.323 | 20.565 | <0.001*** | [6.008, 7.278] |
| **Phosphotau181** | 0.480 | 0.256 | 1.877 | 0.061 | [-0.022, 0.982] |
| **Sex** | 0.358 | 0.054 | 6.603 | <0.001*** | [0.251, 0.465] |
| **Time (years)** | -0.011 | 0.011 | -1.039 | 0.300 | [-0.032, 0.010] |
| **APOE heterozygote** | -0.103 | 0.062 | -1.650 | 0.100 | [-0.225, 0.019] |
| **APOE homozygote** | -0.062 | 0.187 | -0.333 | 0.739 | [-0.428, 0.303] |
| **SCD diagnosis** | -0.095 | 0.065 | -1.460 | 0.145 | [-0.222, 0.032] |
| **MCI diagnosis** | -0.434 | 0.082 | -5.262 | <0.001*** | [-0.595, -0.273] |
| **AD diagnosis** | -0.802 | 0.137 | -5.846 | <0.001*** | [-1.071, -0.533] |
| **Age** | -0.039 | 0.004 | -8.866 | <0.001*** | [-0.047, -0.030] |
| **Sex × Time** | -0.005 | 0.006 | -0.817 | 0.415 | [-0.017, 0.007] |
| **Time × APOE heterozygote** | -0.006 | 0.007 | -0.879 | 0.380 | [-0.019, 0.007] |
| **Time × APOE homozygote** | -0.044 | 0.019 | -2.288 | 0.023* | [-0.081, -0.006] |
| **Time × SCD** | 0.00003 | 0.007 | 0.004 | 0.997 | [-0.013, 0.013] |
| **Time × MCI** | -0.037 | 0.009 | -4.017 | <0.001*** | [-0.055, -0.018] |
| **Time × AD** | -0.048 | 0.018 | -2.747 | 0.006** | [-0.083, -0.014] |
| **Phospho-tau × Sex** | -0.161 | 0.122 | -1.319 | 0.188 | [-0.401, 0.079] |
| **Phospho-tau × Time** | -0.015 | 0.026 | -0.564 | 0.573 | [-0.066, 0.036] |
| **Phospho-tau × APOE heterozygote** | -0.105 | 0.129 | -0.814 | 0.416 | [-0.358, 0.148] |
| **Phospho-tau × APOE homozygote** | -0.339 | 0.261 | -1.298 | 0.195 | [-0.852, 0.174] |
| **Phospho-tau × SCD** | -0.294 | 0.232 | -1.268 | 0.206 | [-0.749, 0.161] |
| **Phospho-tau × MCI** | -0.221 | 0.238 | -0.930 | 0.353 | [-0.688, 0.246] |
| **Phospho-tau × AD** | -0.257 | 0.262 | -0.979 | 0.328 | [-0.774, 0.260] |
| **Phospho-tau × Sex × Time** | 0.012 | 0.014 | 0.903 | 0.367 | [-0.015, 0.040] |
| **Phospho-tau × Time × APOE heterozygote** | -0.014 | 0.015 | -0.947 | 0.345 | [-0.043, 0.015] |
| **Phospho-tau × Time × APOE homozygote** | 0.039 | 0.028 | 1.423 | 0.156 | [-0.016, 0.094] |
| **Phospho-tau × Time × SCD** | -0.024 | 0.023 | -1.029 | 0.305 | [-0.069, 0.021] |
| **Phospho-tau × Time × MCI** | -0.051 | 0.024 | -2.126 | 0.034* | [-0.098, -0.004] |
| **Phospho-tau × Time × AD** | -0.041 | 0.030 | -1.371 | 0.171 | [-0.099, 0.017] |

**Note.** Std.Error= Standard Error, , t=t value, p=p-value, CI= confidence intervals; CN = Cognitively Normal; SCD = Subjective Cognitive Decline; MCI = Mild Cognitive Impairment; AD = Alzheimer’s Disease. ***Significance codes:*** *** p ≤ 0.001, ** p ≤ 0.01, * p ≤ 0.05, . p ≤ 0.1, (blank) p > 0.1.

### **Table S12. Sensitivity analysis using residual-based TIV correction**

| **Region** | **Predictor** | **Estimate** | **Std. Error** | **df** | **t** | **p-value** |
| --- | --- | --- | --- | --- | --- | --- |
| **Basal forebrain** | Intercept | 0.697 | 0.069 | 710.5 | 10.11 | <0.001 |
|  | APOE ε4 heterozygote | −0.021 | 0.013 | 709.4 | −1.60 | 0.111 |
|  | APOE ε4 homozygote | −0.040 | 0.028 | 707.6 | −1.45 | 0.148 |
|  | Time (years) | 0.0003 | 0.0012 | 9311 | 0.22 | 0.823 |
|  | SCD vs CN | −0.024 | 0.014 | 701.3 | −1.66 | 0.097 |
|  | MCI vs CN | −0.133 | 0.018 | 709.7 | −7.30 | <0.001 |
|  | AD vs CN | −0.248 | 0.024 | 726.9 | −10.52 | <0.001 |
|  | Age at baseline | −0.009 | 0.001 | 710.0 | −9.29 | <0.001 |
|  | Sex × Time | −0.0004 | 0.0011 | 5064 | −0.32 | 0.748 |
|  | Time × APOE ε4 heterozygote | −0.00002 | 0.0013 | 10020 | −0.02 | 0.987 |
|  | Time × APOE ε4 homozygote | −0.00010 | 0.0027 | 9719 | −0.04 | 0.972 |
|  | Time × SCD | −0.00002 | 0.0013 | 10190 | −0.01 | 0.991 |
|  | Time × MCI | 0.00014 | 0.0018 | 9938 | 0.07 | 0.941 |
|  | Time × AD | 0.00039 | 0.0032 | 9390 | 0.12 | 0.901 |
| **Hippocampus** | Intercept | 2.989 | 0.183 | 711.0 | 16.29 | <0.001 |
|  | APOE ε4 heterozygote | −0.094 | 0.035 | 710.8 | −2.67 | 0.008 |
|  | APOE ε4 homozygote | −0.366 | 0.074 | 711.6 | −4.94 | <0.001 |
|  | Time (years) | −0.016 | 0.005 | 592.7 | −3.53 | <0.001 |
|  | SCD vs CN | −0.036 | 0.038 | 711.0 | −0.95 | 0.342 |
|  | MCI vs CN | −0.397 | 0.049 | 712.6 | −8.18 | <0.001 |
|  | AD vs CN | −0.924 | 0.063 | 713.9 | −14.79 | <0.001 |
|  | Age at baseline | −0.039 | 0.003 | 709.9 | −15.14 | <0.001 |
|  | Sex × Time | 0.002 | 0.004 | 608.2 | 0.62 | 0.538 |
|  | Time × APOE ε4 heterozygote | −0.004 | 0.005 | 615.0 | −0.87 | 0.385 |
|  | Time × APOE ε4 homozygote | −0.038 | 0.010 | 622.8 | −3.88 | <0.001 |
|  | Time × SCD | −0.007 | 0.005 | 586.0 | −1.45 | 0.149 |
|  | Time × MCI | −0.051 | 0.006 | 620.7 | −7.97 | <0.001 |
|  | Time × AD | −0.079 | 0.008 | 677.4 | −9.40 | <0.001 |

**Publication bibliography**

Kilimann, Ingo; Grothe, Michel; Heinsen, Helmut; Alho, Eduardo Joaquim Lopez; Grinberg, Lea; Amaro, Edson et al. (2014): Subregional basal forebrain atrophy in Alzheimer's disease: a multicenter study. In *Journal of Alzheimer's disease : JAD* 40 (3), pp. 687–700. DOI: 10.3233/JAD-132345.

Sanchis-Segura, Carla; Ibañez-Gual, Maria Victoria; Adrián-Ventura, Jesús; Aguirre, Naiara; Gómez-Cruz, Álvaro Javie; Avila, César´; Forn, Cristina (2019): Sex differences in gray matter volume: how many and how large are they really? In *Biology of Sex Differences* 10 (32). DOI: 10.1186/s13293-019-0245-7.

Sanchis-Segura, Carla; Ibañez-Gual, Maria Victoria; Aguirre, Naiara; Cruz-Gómez, Álvaro Javier; Forn, Cristina (2020): Effects of different intracranial volume correction methods on univariate sex differences in grey matter volume and multivariate sex prediction. In *Scientific reports* 10 (1), p. 12953. DOI: 10.1038/s41598-020-69361-9.
